# A Multiphase Combined Treatment Including Individualized Multimodal Immunotherapy and Tumor Microenvironment Therapy for Glioblastoma: A Decade of Experience with Real-World Patients

**DOI:** 10.3390/biomedicines14092044

**Published:** 2026-09-11

**Authors:** Linde F. C. Kampers, Jennifer Kosmal, Peter Van de Vliet, Stefaan W. Van Gool

**Affiliations:** Praxis for Immuno-Oncology and Translational Medicine at the Immun-Onkologisches Zentrum Köln, Hohenstaufenring 30-32, 50674 Cologne, Germany

**Keywords:** glioblastoma, tumor microenvironment, individualized multimodal immunotherapy, dendritic cell vaccines, oncolytic virus, modulated electro-hyperthermia, checkpoint inhibitors, quality of life, health utility index

## Abstract

**Background/Objectives**: A potential association between individualized multimodal immunotherapy (IMI) within a multiphase combined treatment strategy and prolonged survival was explored through retrospective real-world data analysis. **Methods**: The patient selection criteria were as follows: treatment after 27 May 2015, adults between ages 18 and 70, GB diagnosis and IDH1wt status documented, known MGMT promoter methylation status (methylated versus unmethylated), known status of OS, and no second malignancy. IMI is currently only accessible to patients with a >60 KPI and sufficient financial resources. **Results**: A total of 104 patients were identified, distributed as per MGMT methylation versus unmethylation status, respectively, as 18% female/25% male versus 23% f/38% m; the median age at intake was 54 y versus 50 y; the resection extent was 12 R0, 28 < R0 and three not documented versus 25, 28 and eight; and the median KPI at intake was 70 overall. Patients received a median of 37 versus 31 modulated electro-hyperthermia sessions, 37 versus 32 NDV injections, and two versus one dendritic cell vaccines. The median OS and percentage 2 y OS were 33.1 months and 69.7% *versus* 19.0 months and 35.4%. There were no major adverse reactions (ARs), but the AR burden increased with checkpoint inhibitor use. A 33-patient subset was analyzed on health-related quality of life (HRQoL) throughout IMI treatment, based on at least five EQ-5D-5L questionnaires available from intake. Mobility and self-care affected HRQoL less than usual activity, pain/discomfort and anxiety/depression. Throughout IMI, HRQoL remained stable. Before progressive disease, the median health utility index was 0.86, resulting in a median quality-adjusted life-months of 4.6 versus a median of 5.4 calendar months. **Conclusions**: Real-world observation indicates IMI may prolong GB patient OS while maintaining HRQoL.

## 1. Introduction

Glioblastoma (GB) remains the most common and lethal brain malignancy in adults. In 2005, chemotherapy (CTx) with temozolomide (TMZ) plus radiation therapy (RTx; together, RCTx) replaced RTx alone in standard-of-care (SoC) treatment, marking significant improvement in the overall survival (OS) of newly diagnosed patients [1]. Since then, it has been shown that GBs with unmethylated O-6-methylguanine-DNA methyltransferase (MGMT) promoters are resistant to TMZ treatment [2]. Despite this insight, SoC has remained unchanged over the last 20 years [3,4], and the median OS has stagnated at only 12–15 months depending on MGMT promoter methylation status. Though GB 1-year OS has risen from 35.0% to 43.0% [5,6,7,8], its location and the blood–brain barrier (BBB) make GB inaccessible for many treatment options. When resection is possible, over 50% of patients experience recurrence within 6 months [9]. 

Immunotherapy adds novel treatment options to current SoC. Immunogenic cell death (ICD) immunotherapy, such as tumor-treating fields (TTFs) [10,11], modulated electro-hyperthermia (mEHT) [12], or oncolytic viruses (OVs) [13,14], induces GB-specific cell death, resulting in a release of tumor antigens into the blood. Active specific immunotherapy, such as dendritic cell (DC) vaccines [15,16], trains the immune system to specifically target GB. Modulatory immunotherapy in the form of immune checkpoint inhibitors (ICIs) [17] blocks tumor-induced T-cell inactivation. Despite promising results when tested in combination [18], immunotherapy is still under development and not part of SoC treatment. The personalized or individualized nature of immunotherapy complicates testing within the standardized format of a randomized clinical trial (RCT). Due to the German legal “compassionate use” or “individueller Heilversuch” framework, patients themselves can choose to undergo treatments that are found safe but have not yet been approved through an RCT. The compassionate use framework is strictly regulated: patients can only opt for treatments under strict medical supervision that are found safe, and they must provide informed consent. Patients undergoing new or combination immunotherapy are heavily monitored, allowing for retrospective analysis of their combined patient data. While this cannot function as definitive proof of treatment efficacy, as it lacks a control group and randomization and might introduce bias due to limited financial availability, it does allow crucial insight into patient health whilst undergoing an immunotherapy combination strategy embedded in their standard-of-care regimen.

**Figure 1:**
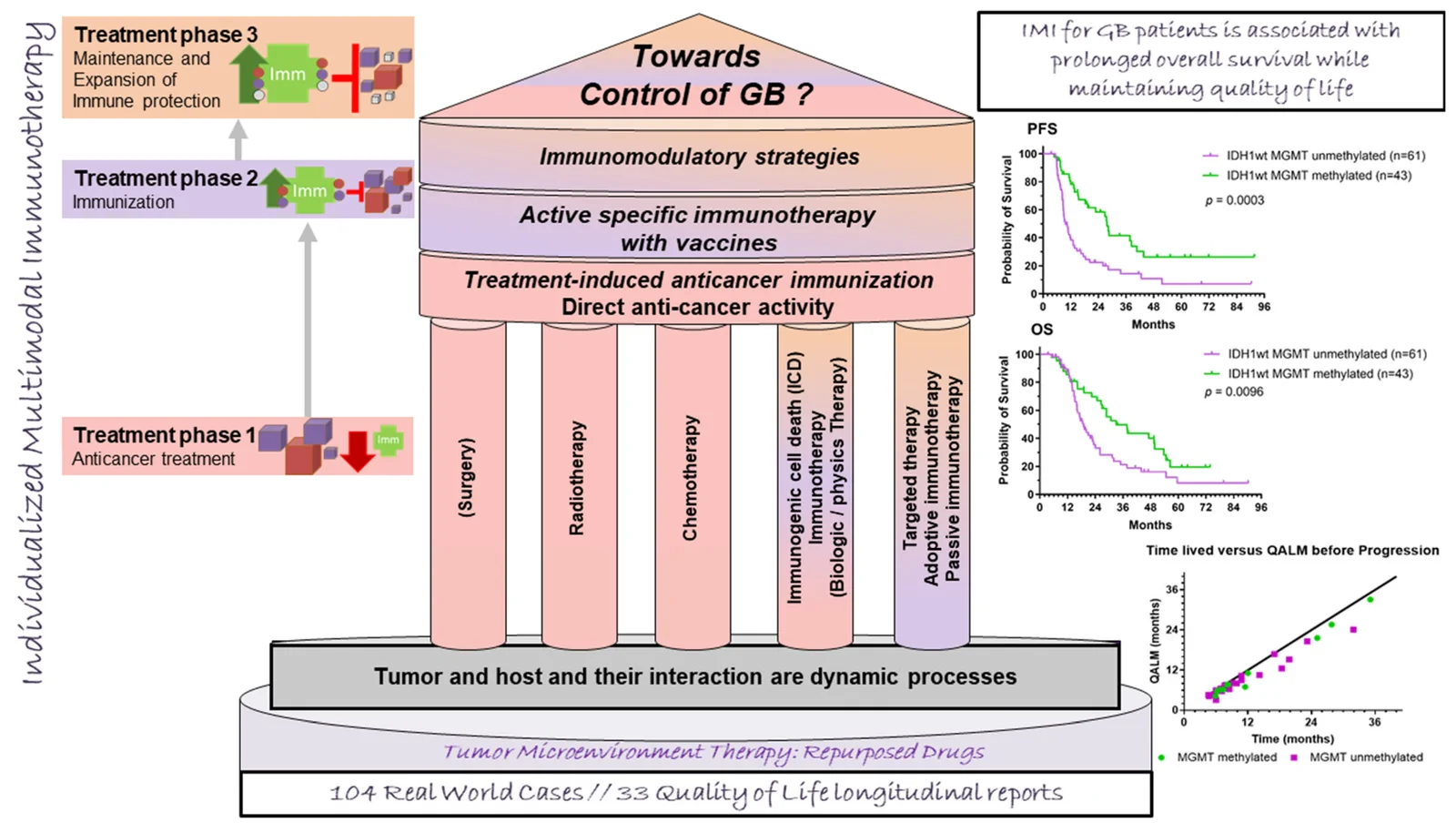
Abstract figure. From (**top left**) to (**bottom right**): The multi-phased individualized multimodal immunotherapy treatment plan is shown. Imm: immune system; green arrow: improvement; red arrow: decline; red T-bar: suppression; blocks of different colors: the tumor with its different subclones. The temple structure represents the rationale behind effective glioblastoma treatment. As the tumor, host and their interaction are highly dynamic, treatment should reflect this. Standard of care (first three pillars) is combined with different types of immunotherapies for control of glioblastoma (fourth and fifth pillars). Colors red, purple and orange correspond to the different treatment phases. Repurposed drugs lay the groundwork for effective anticancer treatment through tumor microenvironment treatment. In this study, 104 real-world patients were treated following this rationale. Of these, 33 longitudinal quality-of-life reports were available. Plots show the associated progression-free and overall survival of the 104 MGMT-methylated (green) and -unmethylated (purple) patients, as well as the cumulative quality-adjusted life months (QALM) of the 33 patients compared with the time lived.

The immunotherapy combination strategy applied here (Figure 1) has been developed over several years, based on the ongoing rapid scientific developments and early clinical trial evidence. Seven key characteristics play a crucial role in the development of GB and its tumor microenvironment (TME): (I) Glioma stem-like cells (GSCs) have the properties of self-renewal and multi-lineage differentiation, lending the tumor resilience to treatment [19]. (II) Constant GB growth causes irregular vascularization, resulting in chronic and cyclic hypoxia [20]. The push and pull of oxygen give rise to reactive oxygen species (ROS) and drive cellular mutation, intra-tumoral heterogeneity and metastasis. (III) Cyclic hypoxia paired with unregulated growth severely impacts the metabolic requirements of tumor cells. As a result, side products of glycolysis and the Krebs cycle are redirected for cytosolic acetyl-CoA synthesis [21]. GB energy generation is redirected towards anaerobic fermentation, and GSCs come to rely heavily on fatty acid oxidation to make up for resulting energy deficiencies. Nucleotide synthesis through de novo and salvage pathways is highly upregulated. (IV) During tumorigenesis, cells enter a constant inflammatory state [22]. The subsequent increase in growth factors, proangiogenic factors, inductive epithelial–mesenchymal transition signals, and survival factors sustains proliferative signaling, promotes vascularization, and inhibits apoptosis. Cross-talk between tumor-associated macrophages (TAMs), neutrophils, myeloid-derived suppressor cells (MDSCs) and T cells turns the GB TME into an immunosuppressive site. Immune cells that do enter the TME adopt an immunosuppressive subtype, which mediates the effects of RTx [23]. In a negative spiral, the metabolic changes in GB and its TME stimulate MDSC recruitment and immunosuppression. The importance of TME expression patterns in GB prognosis was reinforced in the work of Li et al., who demonstrated the prognostic value of mucosa-associated lymphoid tissue translocation gene 1 (MALT1) levels in the TME, with higher levels being implicated in worse prognosis, activating nuclear factor-κB and promoting tumor proliferation and invasion. Such insights into the TME can then be used to find new therapeutic targets [24]. (V) The unique GB environment features a neuron–glioma axis. Direct glutamatergic synaptic input from presynaptic neurons to postsynaptic glioma cells sustains the glioma cell network [25]. (VI) Circadian rhythms affect immune system functioning [26]. Time of day also affects MGMT methylation status [27]. Thus, TMZ efficacy increases with morning administration [28]. (VII) The gut–brain axis is the continuous interaction between the gastrointestinal tract and central nervous system (CNS). Continuous bidirectional communication occurs through neuronal, endocrine and immunological pathways [29]. GB causes dysbiosis [29].

GBs’ high spatio-temporal heterogeneity further explains the difficulty of eradication. No matter what underlying GB mechanism is targeted, treatments applied in isolation have yielded little to no success [19]. The answer to effective anti-GB therapy lies in strategic and carefully timed treatment combination. The research currently done into methods to resensitize GB cells to TMZ [30,31] supports this need. Immunotherapy relies heavily on a functioning immune system. GB creates an immuno-hostile TME and is skilled in immune evasion. In addition, SoC treatment methods further weaken the immune system. This inspired the conceptualization of a three-phase combined treatment strategy including individualized multimodal immunotherapy (IMI) for GB patients. During the first anticancer phase, SoC is combined with ICD immunotherapy, consisting of oncolytic Newcastle Disease Virus (NDV) injections combined with mEHT, to reduce tumor size and burden but also to target GSCs and alter the TME. During the second immunization phase, immune function is restored through active specific immunotherapy with patient-derived DC vaccines (IO-VAC^®^), loaded with patient-derived tumor antigens. In combination with ICD therapy for actual antigen yield and modulatory immunotherapy, consisting of anti-programmed cell death protein ligand 1 (PD-L1) ICIs [32], the immune system is strengthened at a time when the GB is weakened on all fronts. The third maintenance and expansion of immune protection phase consists of further ICD therapies, a boost IO-VAC^®^, long-peptide vaccines and modulatory immunotherapy with the aim of counteracting new GB sub-clones at an early stage, as well as counteracting T-cell exhaustion. In all treatment phases, TME treatments with anti-inflammatory drugs, risedronate, mirtazapine, mebendazol, metformin, atorvastatin, perampanel and melatonin, together with diet instructions, are implemented. Continuous monitoring through liquid biopsy sampling and MRI analysis ensures IMI adaptation where necessary, tailored specifically and continuously to patient needs.

The goal of this research is to study the longitudinal associative effects of IMI embedded within SoC in a real-world setting, with the purpose of informing the scientific and medical community of the effects and complications of individualized and personalized patient care. Clinical evaluation is required to determine actual causality. In 2023, we outlined and published the conception and rationale behind IMI [33]. We now present an update on prior retrospective analysis, presenting data on a group of 104 GB patients who underwent IMI, developed step by step over the last decade, together with TME treatment, which was more recently implemented, as part of first-line treatment. In recent years, we were also able to systematically collect data on the health-related quality of life (HRQoL) of a subgroup of patients under treatment. Till now, there are no data available on the longitudinal HRQoL of GB patients undergoing any form of immunotherapy alongside SoC.

## 2. Patients and Methods

### 2.1. Patients

The IOZK received approval for the production of IO-VAC^®^ on 27 May 2015 (DE_NW_04_MAI_2015_0033), thus used as one of the patient selection criteria. The product IO-VAC^®^ is a specific autologous anticancer DC vaccine for intradermal application: patient-derived autologous DCs differentiated from monocytes and loaded with tumor antigens derived from autologous lysed tumor cells in the presence of immunologic danger signals from NDV. Since then, all patients were treated within the German legal “compassionate use” or “individueller Heilversuch” framework upon providing written informed consent. As immunotherapy relies on an active immune system, patients are required to have a KPI > 60 to qualify for IMI treatment. Data management was established through both an electronic medical dossier and meticulously structured patient medical data recording using a specifically developed patient database using the FileMaker Pro 18 Advanced software (Claris International Inc.). For the current analysis, patient selection criteria were defined prior to data retrieval: 1/ patients initiating IOZK treatment between 27 May 2015 till 1 September 2025; 2/ adults aged 18 to 70 years; 3/ documented diagnosis of GB [34], with documented isocitrate dehydrogenase 1 wild type (IDH1wt) status; 4/ IMI as part of the first-line GB treatment; 5/ known MGMT promoter methylation status; 6/ OS data known; and 7/ no second malignancy or gliomatosis cerebri.

SoC, care for co-morbidities, supportive care and imaging were usually provided by the local oncology center and considered throughout treatment planning at the IOZK. In case of treatment discontinuation, follow-up occurred through mail or phone contact.

### 2.2. Therapy

**Figure 2:**
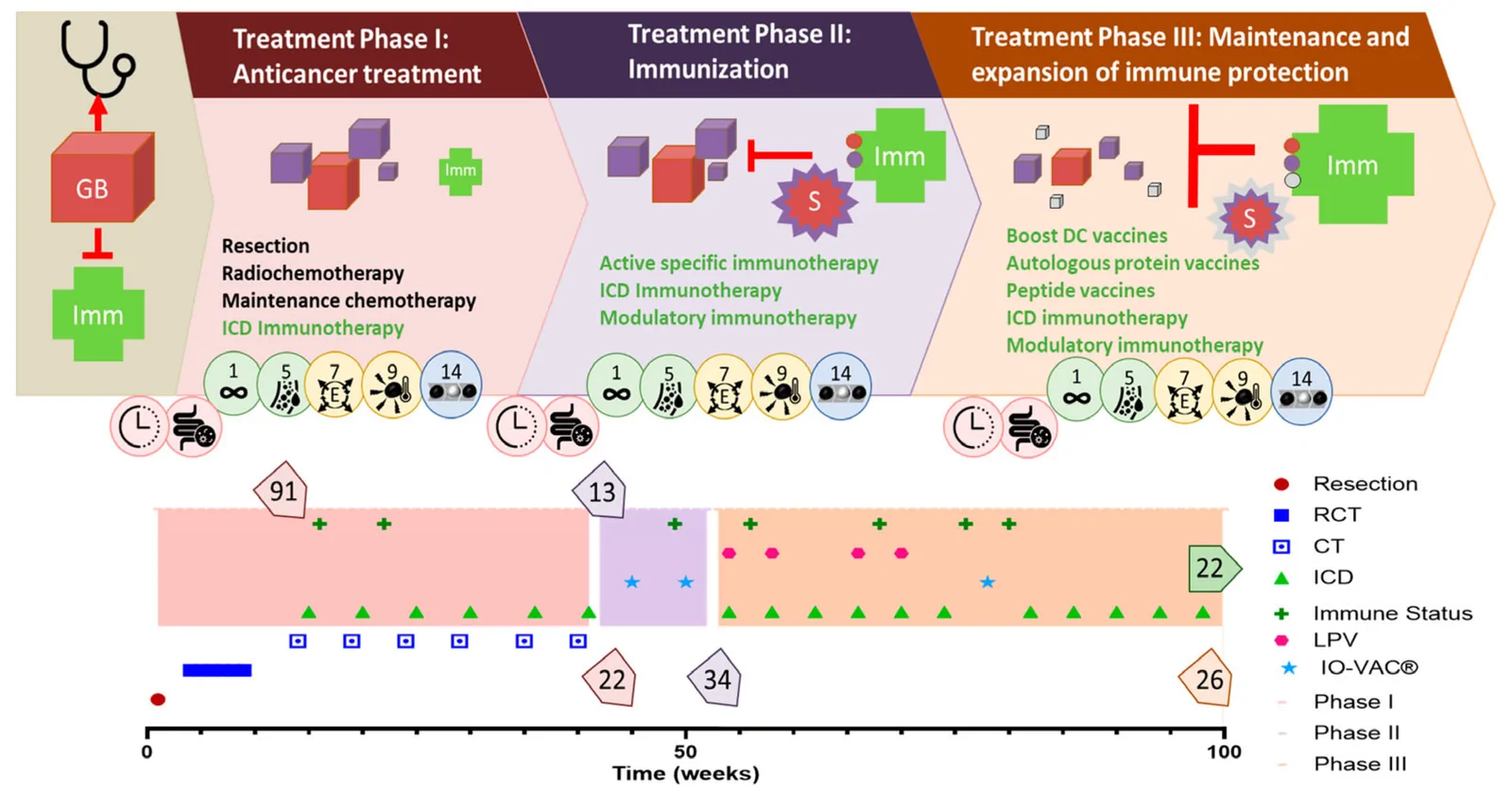
(**Top**): Individualized multimodal immunotherapy (IMI). The multiphase treatment regimen is outlined from first diagnosis to anticancer treatment and immunization to maintenance and expansion of immune protection. Tumor is represented by blocks, with mutated derivatives indicated by color changes. Hallmarks of cancer combatted are indicated by numbered icons (numbers referring to specific hallmarks of cancer [19]) per treatment phase. Chrono-immunotherapy and gut–brain axis are also included as icons. GB: glioblastoma; Imm: immune system; ICD: immunogenic cell death; S: serum containing tumor-specific antigens (indicated in different colors matching tumor mutations); DC: dendritic cell. Figure adapted from Kampers et al.’s study [19]. (**Bottom**): IMI strategy fit. Colors match the treatment phase and overlap with treatments that are part of IMI. Numbers inserted with arrows indicate influx and outflow of patients at different stages of the proposed strategy, dependent on individual choices, well-being, and abilities. The green arrow indicates patients actively undergoing treatment.

The concept of IMI was developed and continuously re-evaluated over a ten-year period (Figure 2). The rationale behind each type of repurposed drug was extensively addressed in a previous review [19]. The decision to separate DC vaccination from maintenance CTx led to the development of a multiphase global strategic treatment plan. During anticancer treatment phase I, SoC maintenance CTx cycles are strengthened with ICD immunotherapy. The ICD therapy occurs two to three days after the 5-day TMZ or 6-day lomustin/TMZ maintenance course, which initially covered 3 days and, since 2017, now covers 5 consecutive days. ICD therapy consists of a daily pretreatment of 50 to 100 mL of blood with 30 µg/mL ozone therapy and an infusion with 100 mL of Mannitol 15%, followed by a bolus intravenous injection of 10 × 10^7^ NDV infectious particles of the mesogenic oncolytic MTH-68 strain, diluted in 1 mL of 0.9% NaCl containing 0.5% of a 0.5 M EDTA solution [35]. After each daily NDV injection, the patient undergoes mEHT, using a standard-sized antenna of the Oncothermia EHY-2000 device (www.oncotherm.com; accessed on 23 January 2026). In 2015, the dose was set at 40 Watt for 50 min. In 2018, the mEHT dosage was increased to 60 Watt if tolerated. During mEHT, patients receive intravenous vitamin C in a dose of 7.5 g initially and later 15 g, combined with 40 mg of MgCl_2_, 45 mg of CaCl_2_, 15 mg of KCl, 10 mg of Magnesiocard, 5 mL of Nervoregin, and Selenase (dose adjusted to current patient-specific selenium blood levels).

Three to four weeks after anticancer phase I, a second immunization phase is started. Patients receive two consecutive 8-day IO-VAC^®^ DC vaccination cycles with a three- to four-week interval. Autologous DCs were differentiated from plastic-adherent monocytes over 5 days in the presence of 800 U/mL IL-4 and 1000 U/mL GM-CSF. During this time, the patient undergoes ICD therapy. Tumor antigens either consist of tumor proteins derived from tumor tissue lysates or ICD therapy-induced serum-derived antigenic extracellular microvesicles and apoptotic bodies. The antigen-loaded DCs are matured in the presence of 1 × 10^5^ infectious NDV particles per million DCs, and 1900 U/mL IL-1β, 1100 U/mL TNF-α and 1000 U/mL IL-6. All cytokines were purchased from Cellgenix (Freiburg, Germany). After quality control, all loaded mature DCs are taken up in 6 mL of Ringer’s solution and administered intradermally in two separate 3 mL doses. Since 2024, the immunotherapeutic effect of the active specific immunotherapy with IO-VAC^®^ has been modulated by one dose of 1680 mg of atezolizumab, based on data from Sprooten et al.’s study [36].

After anticancer phase I and immunization phase II, the third maintenance/expansion phase is initiated, consisting of further 5-day ICD treatment courses. Since 2021, long peptide vaccines of 600 µg of WT1 peptide mix (3 × 200 µg) and 300 µg of survivin (JPT, Berlin, Germany) have been included in consecutive ICD maintenance treatments. Peptide vaccines are administered intradermally twice in skin primed with imiquimod (applied 12 h before and 12 h and 36 h after vaccination), combined with local intradermal addition of 0.3 mL of NDV. Since 2024, low-dose ICI nivolumab (anti-programmed cell death protein 1 (PD-1)) and ipilimumab (anti-cytotoxic T-lymphocyte-associated protein 4 (CTLA-4)) have been used according to a published schedule [37].

The majority of patients were taking complementary drugs and supplements, in part guided and prescribed by the local oncologist, but also frequently on the initiative of the patient and their caretakers. Since 2024, a more structured approach of a mixture of repurposed and complementary drugs focused on seven particular cancer domains has been gradually implemented [19]. Patients received antidepressant drugs in the context of GSCs. Antidepressants consisted mostly of dose-escalated mirtazapine (15 mg to 30 mg to 45 mg), or alternatively imipramine or fluoxetine. In the context of hypoxia, 2 × 500 mg of mebendazole was proposed. The domain of anti-inflammation is tackled through four different approaches: (1) NF-κB blockade with curcumin oral spray [38,39] (Apurano Pharmaceuticals GmbH, Warngau, Germany); (2) Cox-2 inhibition with 2 × 200 mg of celecoxib per day; (3) histamine receptor 1 blockade with 1 to 2 mg of clemastine per day; and (4) driving tumor-associated macrophage from the immunosuppressive M2 to the anti-inflammatory M1 phenotype with 35 mg of risedronate once per week (with a mandatory dental check prior to treatment start). Metabolic dysregulation was treated with 2 × 500 mg of metformin per day and 10 mg of atorvastatin, with dose escalation to 40 mg per day. The neuron–glioma axis was blocked by daily doses of perampanel (with dose escalation from 2 mg to 8 mg). For the psycho–neuro–endocrino–immunology axis, 20 mg of melatonin per day was proposed, and CBD oils (without THC) and 4.5 mg of low-dose naltrexone were allowed. Finally, for gut–brain axis improvement, low-carbohydrate-containing, fiber-rich foods and probiotics were recommended. The use, dosage and combination of treatment-complementary drugs and supplements were decided upon with and after receiving the final informed consent of each individual patient, taking their personal tumor characteristics and statuses, lifestyles, and anticancer treatment plans into account.

### 2.3. Clinical Course Assessment

For each individual patient, the full clinical course was outlined. The clinical course was based on patient records kept in the FileMaker Pro 18 Advanced software (Claris International Inc.), patient treatment schedules via the Q-MED Omni7 software (Schwerdtner Medizin-Software GmbH), and patient correspondence or correspondence with their primary caregiver or a relative. Patient clinical course included all treatments given at the IOZK facility, as well as information on GB-related surgeries, level of resection when known, imaging results and their reported outcomes. RTx or CTx treatments, as well as treatments in other facilities, were not included in the reported clinical course.

### 2.4. Quality-of-Life Assessment

To assess patient health-related quality of life (HRQoL), the treating physician reports the Karnofsky performance index (KPI) [40] during each appointment. Since October 2021, the EQ-5D-5L questionnaire has been included at the time of each treatment [41] (https://euroqol.org, accessed on 30 January 2026). The EQ-5D-5L consists of a self-reported EQ-5D descriptive system and EQ visual analog scale (EQ-VAS). Licenses for 15 languages were available. Data for the reference health state were derived from mean values for Belgium, the Netherlands and England due to the accessibility of validated value translation. Health utility indices (HUIs) *per* patient *per* measurement were calculated. The time interval between each consecutive questionnaire was translated to quality-adjusted life months (QALM) and the quality months of life lost (QMLL) using the corresponding HUIs. From the initial 104 patients, a subgroup was selected for in-depth HRQoL analysis based on the following criteria: availability of 1/ HRQoL data at the time of the first intake contact at the IOZK, and 2/ at least 5 HRQoL measurements.

### 2.5. Laboratory Tests

Routine laboratory tests were performed by Laborunion in North Rhine-Westphalia (www.laborunion.de; accessed on 23 January 2026). Other tests were performed at Synlab (www.synlab.de; accessed on 23 January 2026). Quantitative and qualitative immune tests were performed in-house, including flow cytometry immunophenotyping of lymphocytes and Stat5 intracellular production assessment to establish T-cell proliferation upon CD3/CD28 or PHA stimulation [42]. The presence and activity of CD4+ and CD8+ cells were assessed with flow cytometry through IFN-γ, IL-4 and IL-17 production upon stimulation with ionomycin and PMA, adding monensin to inhibit cytokine secretion. NK cell cytotoxicity was established by flow cytometric analysis of stained K562 cells after NK cell exposure with or without IL2 stimulation [43]. Total peroxide and anti-oxidative capacity were determined with the PerOx (TOS/TOC) kit (Immundiagnostik AG, Bensheim, Germany). The presence and chemo-sensitivity testing of circulating cancer cells (CCCs), defined as circulating large cells, not passing a mesh, with mRNA expression of telomerase, ERBB2, c-KT or EGFR above a cut-off ratio of 2 in comparison with the mRNA expression of GAPDH, was assessed by the Biofocus Molecular Oncology Department (www.biofocus.de/molecular-oncology; accessed on 23 January 2026). Since May 2022, circulating tumor DNA in blood has been explored at the time of intake. Cell-free DNA was extracted from 2 PAXgene Blood ccfDNA tubes and analyzed in-house using a next-generation sequencing 19-gene panel with a variant allele frequency (VAF) detection limit of 0.25% [44].

### 2.6. Statistics

Statistics were performed using GraphPad Prism version 10 for Windows, GraphPad Software, La Jolla, CA, USA (www.graphpad.com; accessed on 23 January 2026).

## 3. Results

### 3.1. Patient Characteristics at Individualized Multimodal Immunotherapy Initiation

**Figure 3:**
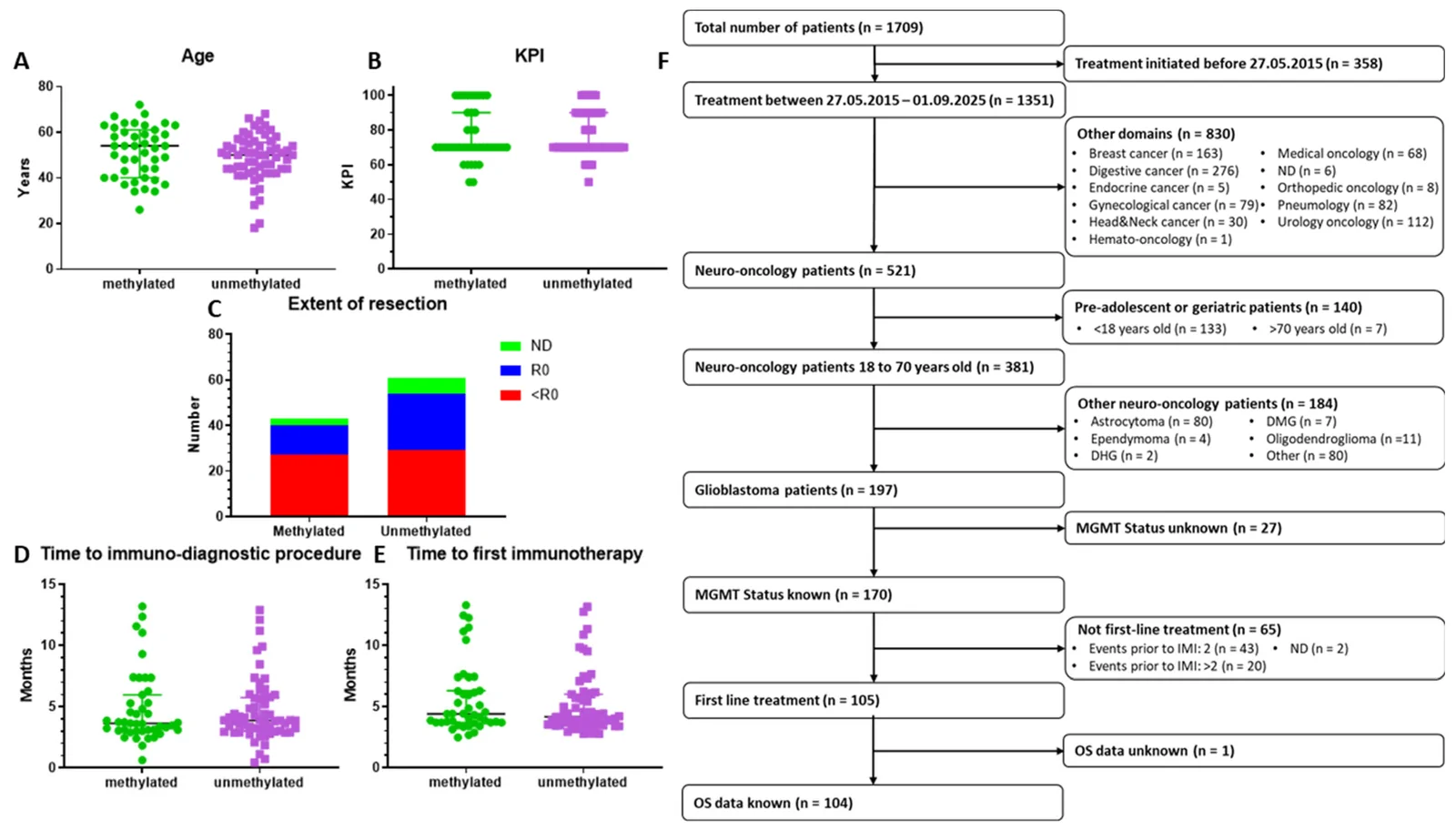
Patient distribution per MGMT promoter methylation status. MGMT-methylated group is depicted in green, and unmethylated group in purple. In each graph except (**C**,**F**), the median and distribution are shown. Values represent status at the time of intake. (**A**) Age; (**B**) KPI: Karnofsky performance index; (**C**) extent of resection (R0: complete resection, <R0: incomplete resection, and ND: not defined); (**D**) time from first diagnosis to immuno-diagnostic procedure; (**E**) time from first diagnosis to first immunotherapy; and (**F**) patient selection overview.

Between 27 May 2015 and 1 September 2025, 521 patients categorized in the domain of neuro-oncology started therapy at the IOZK (Figure 3). Patient inclusion is outlined in Figure 3F.

Of the resulting 104 patients taken into this analysis, 43 patients had MGMT-promoter-methylated (meth or mMGMT) status, while 61 patients were categorized as MGMT-promoter-unmethylated (unmeth or uMGMT). Patients came from 19 different countries: the United Kingdom (28), Poland (22), Germany (11), Austria (5), Hungary (5), Belgium (5), Italy (4), the Netherlands (4), the United States (4), Romania (3), Scotland (2), Ukraine (2), Croatia (2), Sweden (2), Australia (1), Jordan (1), Canada (1), South Africa (1) and Spain (1). The female/male ratio in the meth group was 18/25. The median age at diagnosis was 54 years (y; range: 26 y to 72 y) (Figure 3A). The median KPI at IOZK intake was 70 (range: 50 to 100, Figure 3B). The extent of resection was complete for 13 patients, incomplete for 27 patients, and unknown for three patients(Figure 3C). For the unmeth patients, the female/male ratio was 23/38. The median age was 50 y (range: 18 y to 68 y) (Figure 3A). The median KPI at IOZK intake was 70 (range: 50 to 100, Figure 3B).The number of patients with complete, less than complete and not described extents of resection were 25, 29 and seven, respectively (Figure 3C). Overall, the population distributions between meth and unmeth patients were remarkably similar. Over the ten years of IMI, there was a slight but not significant increase in age at the time of diagnosis of the patients starting immunotherapy. When comparing the first 5 years with the last 5 years, the relative proportion of extent of resection did not differ. The patient KPI at intake for IMI, however, significantly decreased over the ten-year period (meth: *p* = 0.0034; unmeth: *p* = 0.0016).

Most patients came for an initial immune-diagnostic evaluation after completion of RCTx, before or early during the maintenance CTx (Figure 3D,E). The median time between resection and immune-diagnostic evaluation was 3.6 months (m; range: 0.6 m to 13.2 m) for meth patients and 3.9 m (range: 0.4 m to 12.8 m) for unmeth patients. As a consequence, patient IMI initiation varied between prior to SoC (n = 2 meth; 4 unmeth), at the start of or during maintenance CTx (n = 37 meth; 54 unmeth), and post-SoC completion (n = 4 meth; 4 unmeth).

Prior to IMI initiation, patients’ overall immunological status was evaluated via blood analysis (Table 1). First, values were categorized to account for the changes due to continued development over the course of ten years of IMI, both in laboratories and standard ranges. The distribution of all blood analyses, depicted as within range or out of bounds, did not differ between meth and unmeth patients. In general, as expected in GB patients, lymphocyte, T cell, B cell and NK cell levels were low. In more than 60% of the patient population, NK cell function was low as well. A high total anti-oxidative capacity was observed in 85% of patients, while the total peroxide load was increased in 46%. The presence of CCCs was investigated in 102 out of 104 immune-diagnostic samples. More than half of the patients did not have observable levels of CCCs. The majority of CCCs detected did not display elevated mRNA expression of PD-L1. Measurements of MGMT mRNA expression levels are available in 94 immune-diagnostic samples and should reflect MGMT promoter methylation status. In patients characterized as MGMT-promoter-methylated upon intake based on tumor material from resection or biopsy, a slight majority showed elevated levels of MGMT mRNA expression. In the unmeth patient group, a slight majority showed no measurable MGMT mRNA expression levels.

### 3.2. Treatments

**Figure 4:**
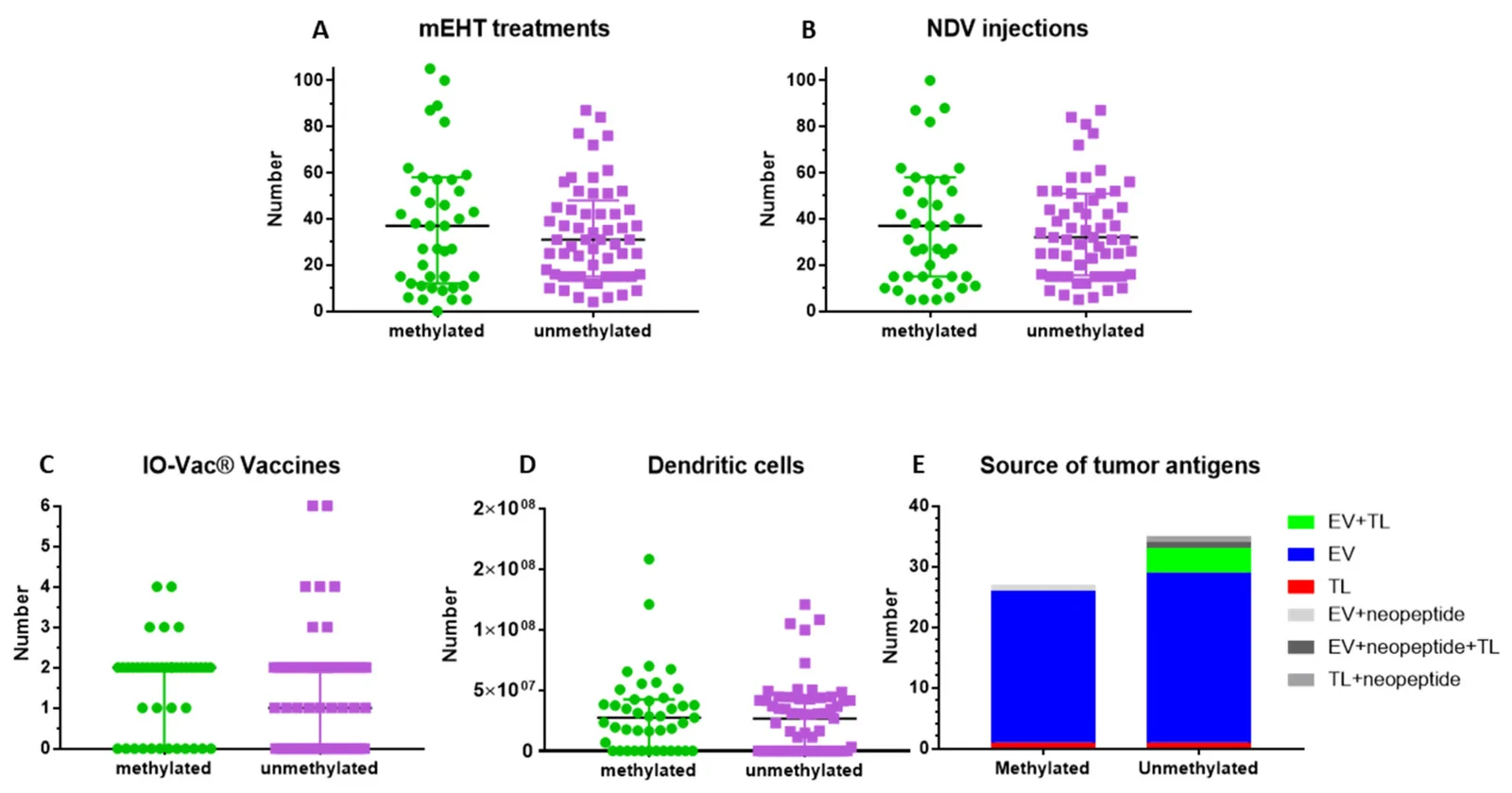
Patient treatment regimen per MGMT promoter methylation status. MGMT-methylated group is depicted in green, and unmethylated group in purple. In each graph except (**E**), the median and distribution are shown. (**A**) Number of mEHT: modulated electro-hyperthermia treatments; (**B**) number of NDV: Newcastle disease virus injections; (**C**) number of IO-VAC^®^ vaccines, dendritic cell therapy; (**D**) median number of dendritic cells given per patient treated with IO-VAC^®^; and (**E**) source of tumor antigens used for IO-VAC^®^ preparation. EV: extracellular vesicles from serum; TL: tumor lysate.

**Figure 5:**
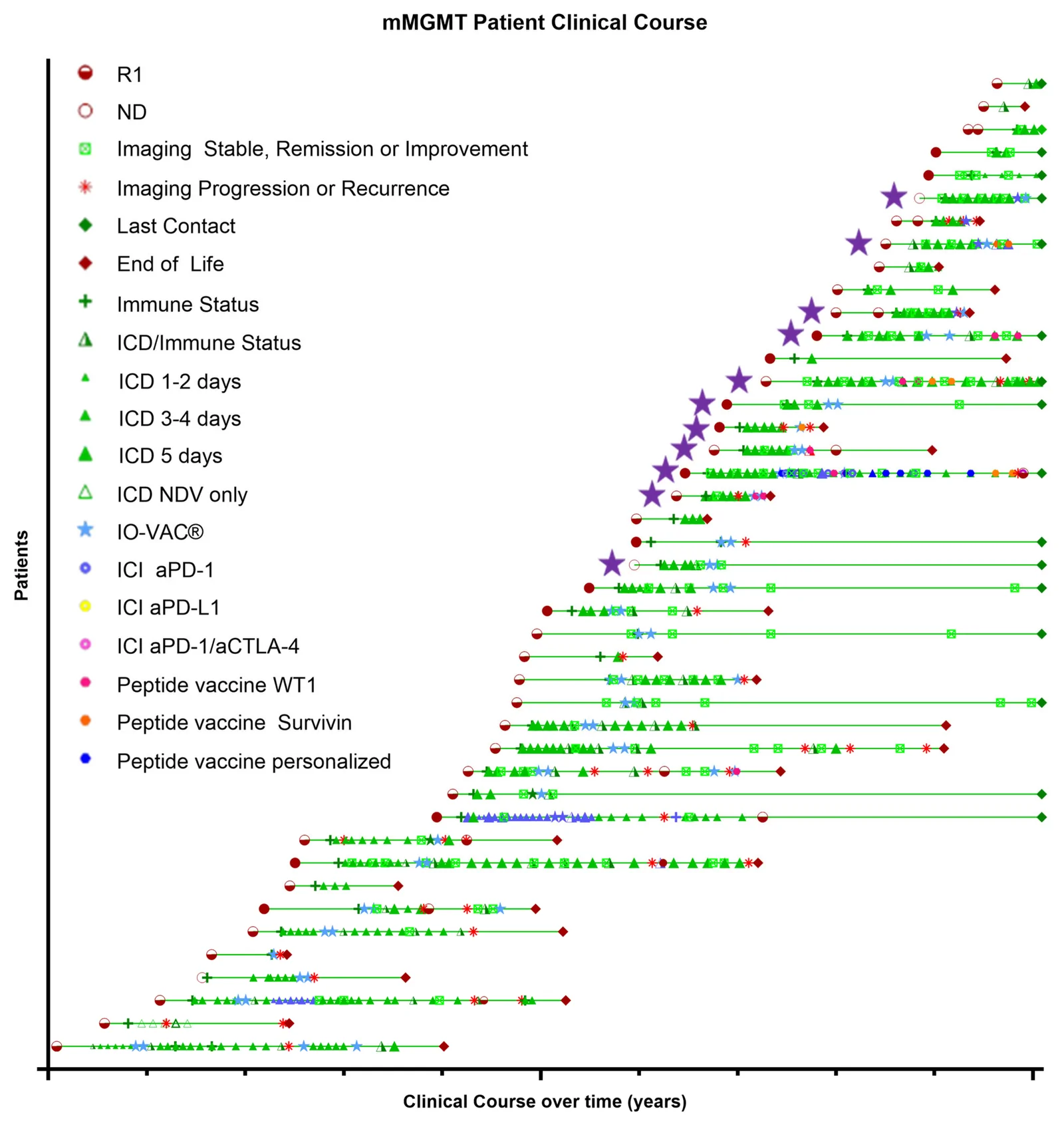
Patient-specific clinical course of MGMT-methylated patients. Level of resection, progression, imaging monitoring, overall survival and IMI treatments are depicted over time and specified in the figure legends. Imaging and surgery were not performed at our facility; if notified, results were depicted as reported by the primary caregiver. Treatments shown only include those provided at our facility. Purple stars placed before a clinical course indicate this patient could be included in the HRQoL analysis.

**Figure 6:**
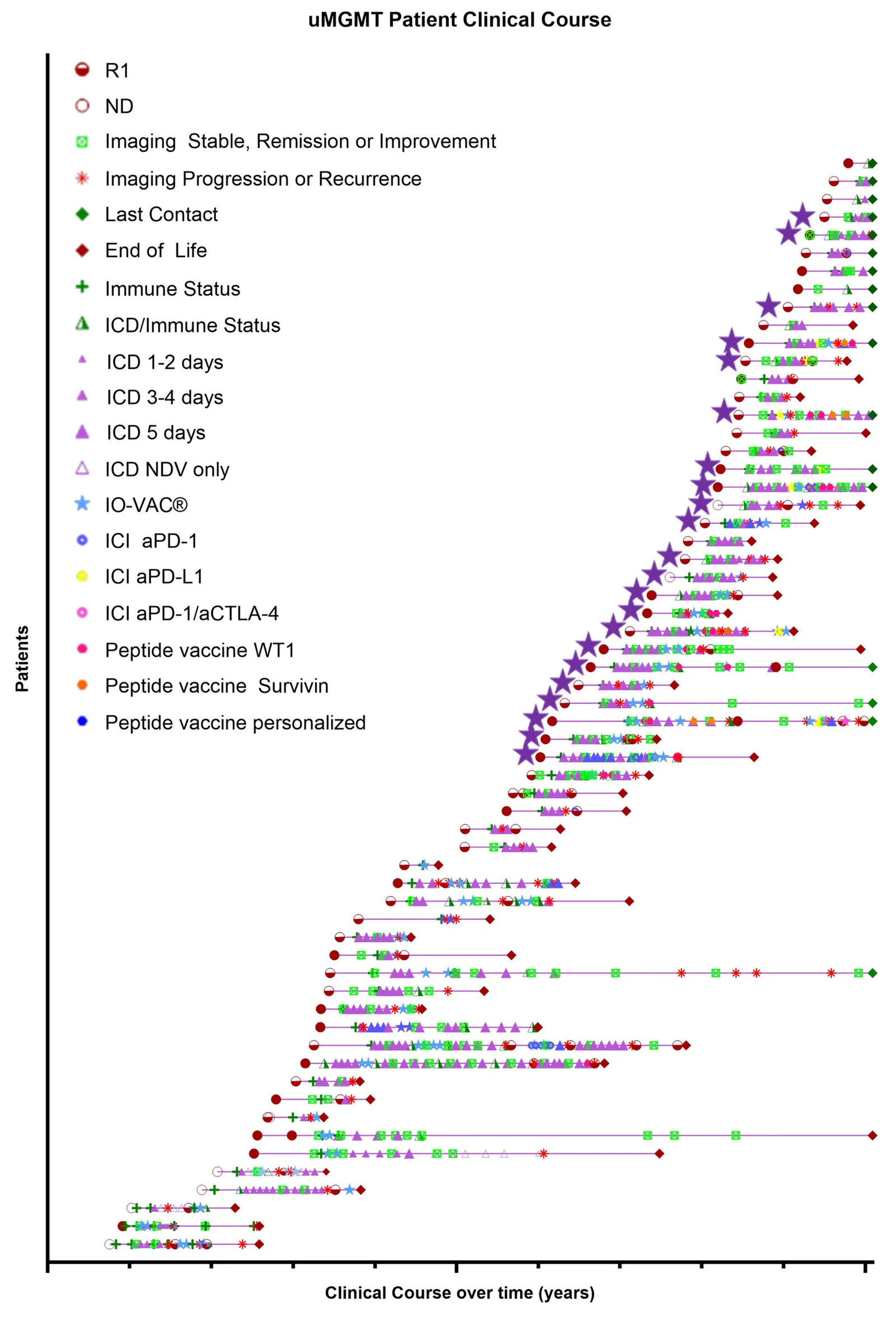
Patient-specific clinical course of MGMT-unmethylated patients. Level of resection, progression, imaging monitoring, overall survival and IMI treatments are depicted over time and specified in the figure legends. Imaging and surgery were not performed at our facility; if notified, results were depicted as reported by the primary caregiver. Treatments shown only include those provided at our facility. Purple stars placed before a clinical course indicate this patient could be included in the HRQoL analysis.

The details of the IMI provided to the patients are shown in Figure 3, Figure 4, Figure 5 and Figure 6, presented separately for meth and unmeth patients. The date of the first event (FE) was defined as the date of tumor resection or biopsy. IMI started at a median of 4.4 m (range: 2.48 to 13.29) and 4.17 m (range: 2.78 to 13.16) after diagnosis in meth and unmeth patients, respectively (Figure 3E).None of the treatment components had statistical differences between patient groups. The patients received a median of 37 (range: 0 to 138) and 31 (range: 4 to 147) mEHT sessions, respectively (Figure 4A); a median of 37 (range: 5 to 138) and 32 (range: 5 to 147) NDV injections, respectively (Figure 4B); and a median of two (range: 0 to 4) and one (range: 0 to 6) DC vaccines, respectively (Figure 4C), with totals of 27.2 × 10^6^ (range: 0 to 158 × 10^6^) and 26.4 × 10^6^ (range: 0 to 120.58 × 10^6^) DCs, respectively (Figure 4D). In the majority of the IO-VAC^®^ products in both meth and unmeth patients, serum-derived tumor secretome components were used as a source of tumor antigens (Figure 4E). Rarely, tumor lysate and/or tumor-specific neopeptides were used. The intake of complementary drugs and supplements was heterogeneous across patients.

Starting in 2024, 1680 mg of atezolizumab (anti-PD-L1) was included at the time of the first IO-VAC^®^. During the maintenance/expansion phase III of IMI, four cycles of low-dose ICIs were added to the maintenance 5-day ICD therapies: 1 mg/kg nivolumab + 0.6 mg/kg ipilimumab in month 1, 0.5 mg/kg nivolumab in month 2, and no ICIs in month 3, though personalized treatment differences occurred between patients.

The clinical course of IMI treatments of each patient shows the large variety between patients undergoing IMI in a real-world setting (Figure 5 and Figure 6). The introduction of each new treatment aspect can be observed. Patient- or primary-caregiver-reported imaging data were included to show tumor status over time. Of the 104 patients, 73 experienced a progression during IMI treatment. Of these, 62 were deceased by the time of writing. The median factor of time between first progression and death/last contact over FE to progression was 0.62 (min. 0.05 to max. 5.42), indicating that patients undergoing IMI survive 0.6 times as long after experiencing progression as from FE to progression.

### 3.3. Survival

**Figure 7:**
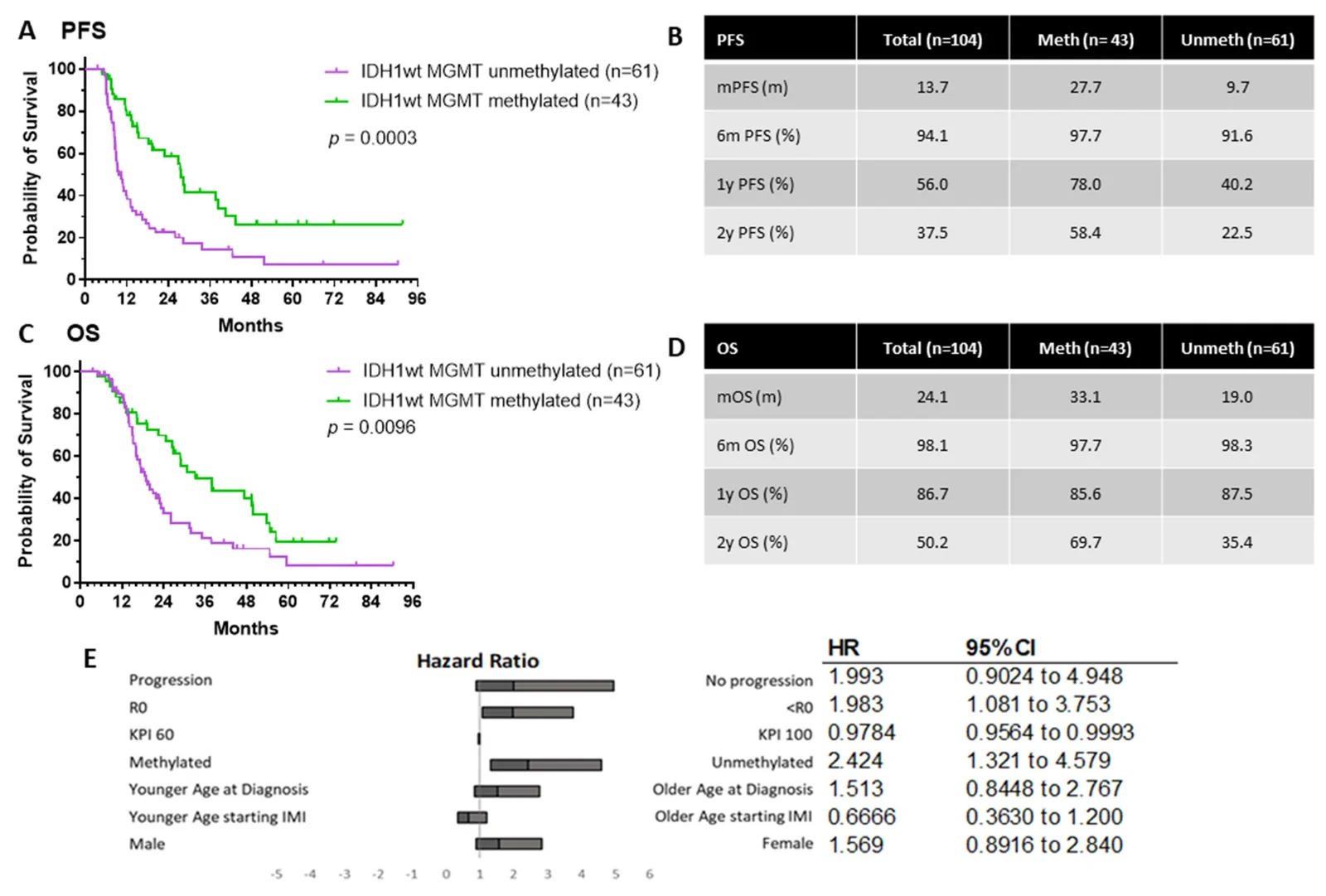
Overview of GB patient survival under individualized multimodal immunotherapy, depicted per patient subtype of MGMT-unmethylated (pink) and MGMT-methylated (green). Level of significance is shown for each graph. (**A**) Progression-free survival (PFS); (**B**) PFS overview; (**C**) overall survival (OS); and (**D**) OS overview. M: month, y: year, and n: number of patients. (**E**) Cox regression analysis showing the hazard ratio with 95% confidence interval (CI) for different factors.

Figure 7 shows patient progression-free survival (PFS) and OS under IMI treatment. Repeated MRI monitoring of tumor development was organized by patients’ primary local neuro-oncology teams. A differential diagnosis between progression versus pseudoprogression remains challenging. Progression was set to the date a radiology report univocally mentioned progressive disease or when the primary oncology team changed the treatment strategy as a consequence of MRI interpretation. Information was available for all patients (Figure 7A,B). The Kaplan–Meier curve showed a median PFS of 13.7 m with a 6 m PFS of 94.1% (confidence interval (CI95%): +3.2, −6.8), a 1 y PFS of 56.0% (CI95%: +9.2, −10.4) and a 2 y PFS of 37.5% (CI95%: +9.9, −9.9). Meth patients had a significantly better PFS than the unmeth patients (log-rank test: *p* = 0.0003). The median, 6 m, 1 y and 2 y PFS values were 27.7 m, 97.7% (CI95%: +2.0, −13.1), 78.0% (CI95%: +9.9, −16.1) and 58.4% (CI95%: +13.9, −17.3), respectively, and 9.7 m, 91.6% (CI95%: +4.8, −10.6), 40.2% (CI95%: +12.4, −12.8) and 22.5% (CI95%: +12.0, −10.1).

As per the selection criteria, OS data were available for all 104 patients (Figure 7C,D). The data on median OS, 6 m OS, 1 y OS and 2 y OS are summarized for the total population: the median OS was 24.1 m, the 6 m OS was 98.1% (CI95%: +1.5, −5.6), the 1 y OS was 86.7% (CI95%: +5.4, −8.5), and the 2 y OS was 50.2% (CI95%: +9.8, −10.8). The OS was significantly better in meth patients (log-rank test: *p* = 0.0096). The median OS, 6 m OS, 1 y OS and 2 y OS for meth patients were 33.1 m, 97.7% (CI95%: +2.0, −13.1), 85.6% (CI95%: +7.7, −15.0) and 69.7% (CI95%: +11.9, −17.0). The median OS, 6 m OS, 1 y OS and 2 y OS for unmeth patients were 19.0 m, 98.3% (CI95%: +1.4, −9.6), 87.5% (CI95%: +6.5, −12.0), and 35.4% (CI95%: +13.2, −13.0). At the time of immune-diagnostic procedures, CCCs were detected in 38 patients, 20 of them being MGMT mRNA-negative and 18 being MGMT mRNA-positive. OS was not significantly different between these patient subgroups.

The Cox regression analysis shows that, within this patient group, MGMT methylation status and level of resection were the only statistically significant predictive factors for prolonged overall survival (Figure 7E).

### 3.4. Adverse Reactions

Among the 104 patients, 29 reported a total of 52 ARs (Table 2), of which eight were grade 3, one grade 4 and one grade 5, assessed by the Common Terminology Criteria for Adverse Events (CTCAE) version 5.0 [45]. The latter was a lung embolism occurring in a wheelchair-bound patient with progressive disease under a therapeutic dose of steroids and bevacizumab treatment. The patient was undergoing the first round of ICD maintenance immunotherapy after two IO-VAC^®^ vaccines. A total of 81% of the ARs were grade 1 or 2. ARs were noticed in the domains of 1/ gastrointestinal disorders (7), 2/ general disorders (10), 3/ hepatobiliary disorders (3), 4/ injury, poisoning and procedural complications (1), 5/ musculoskeletal and connective tissue disorders (2), 6/ nervous system disorders (19), 7/ psychiatric disorders (1), 8/ respiratory, thoracic and mediastinal disorders (1), 9/ skin and subcutaneous tissue disorders (7), and 10/ vascular disorders (1). Twenty-one ARs were connected to an ICD treatment in combination with chemotherapy, 19 ARs could be linked to an IO-VAC^®^ DC vaccination course and 12 ARs appeared during maintenance immunotherapy. In 14 cases, ARs were related to the use of ICIs: colitis (5), flu-like symptoms (3), hepatobiliary disorders (1), arthritis (1), encephalopathy (1), pneumonitis (1), and skin symptoms (2). One grade 1 hepatic failure was connected to the use of repurposed drugs. The mEHT treatment was considered a cause of grade 1 headache in two patients and localized edema in one patient. Further ARs could not be directly linked to IMI intervention. Twice, patients needed hospitalization due to AR. Hospitalizations due to general disease-related adverse events were more frequent.

### 3.5. Health-Related Quality of Life

**Figure 8:**
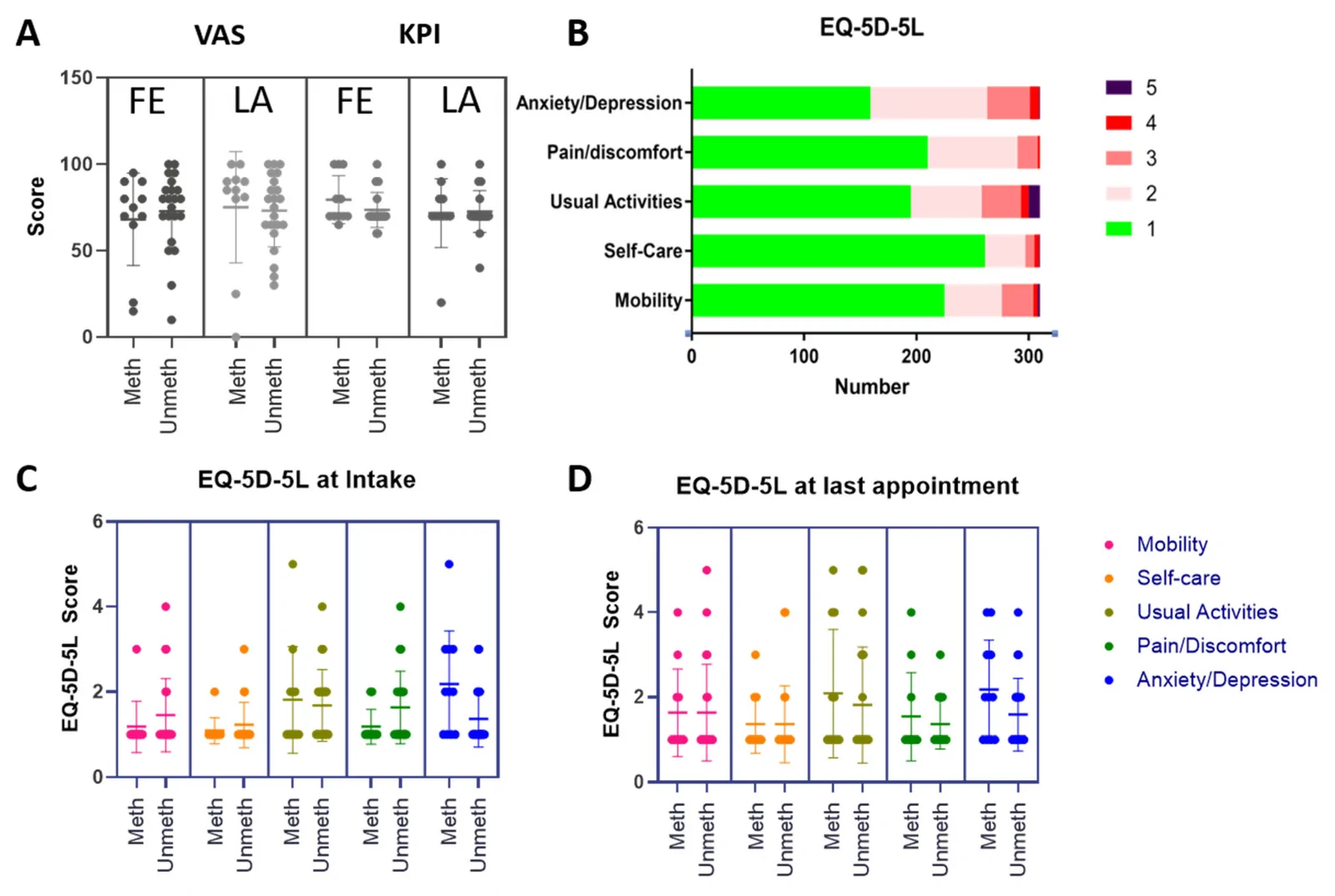
Patient health-related quality of life (HRQoL) assessments. MGMT methylation status, distribution and median are indicated in (**A**,**C**,**D**). (**A**) Comparison between median self-reported VAS (visual analog scale assessment) and physician-evaluated KPI (Karnofsky performance index) at first event (FE) and last appointment (LA). (**B**) Results from EQ-5D-5L questionnaires are depicted as the number of scores per level per dimension. Coloration from green to dark red indicates good to poor ability within a category. (**C**) EQ-5D-5L scores per patient group (meth/unmeth) per dimension at time of intake. (**D**) EQ-5D-5L scores per patient group (meth/unmeth) per dimension at last appointment.

Based on clinical risk factors and nationalities, a representative group of 33 patients (32%) was included for HRQoL analysis (indicated with purple stars in Figure 5 and Figure 6). The median age of this group was 51 y (range: 26 to 68). The female/male ratio was 33% versus 66%. The median KPI was 70 (range: 60 to 100). The extent of resection was documented as 39% R0, 48% <R0 and 12% ND. The included patients came from 11 countries. The response rate from the patients to the questionnaires, given at each treatment course, was 96%. In total, data from 310 questionnaires were sampled. Considering the KPI and EQ-VAS data at the time of the first contact and at the time of the last contact, functioning remained stable during IMI treatment (Figure 8A). There was no difference between meth and unmeth patients. Over the course of treatment, more varied functioning was reflected in the patient-reported EQ-VAS, compared with the treating-physician-assessed KPI. Looking at all the HRQoL EQ-5D-5L reports collected throughout treatment (Figure 8B), the domain of mobility and self-care was considered less problematic in comparison with the domains of usual activity, pain/discomfort and anxiety/depression (chi-square: *p* < 0.0001). Loss of HRQoL was most reported in the domains of usual activity and anxiety/depression. Level 5 was most noticed in the domain of usual activity but absent in the domain of pain/discomfort. Comparing EQ-5D-5L scores between intake and the last appointment displayed a noteworthy stability in patient answers, with no significant changes in any domains and independent of tumor MGMT methylation status (Figure 8C,D).

**Figure 9:**
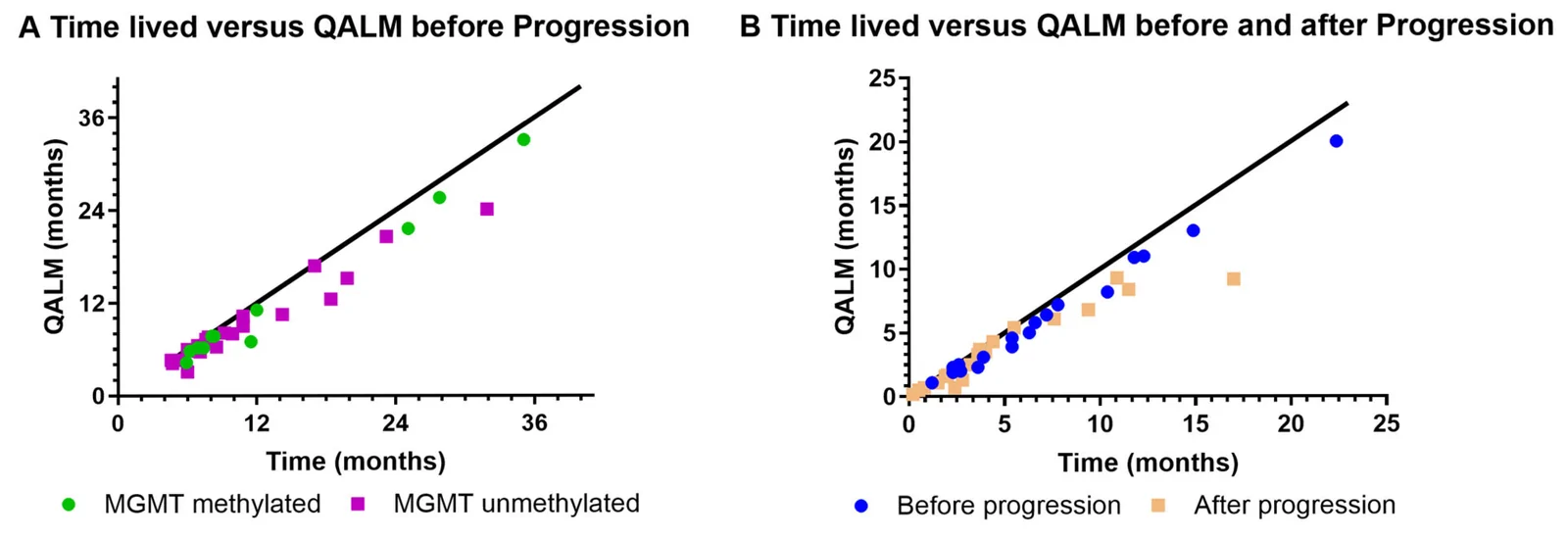
Quality-adjusted life months (QALM in months) versus time lived (months). Comparisons between (**A**) MGMT-methylated (green circles) and MGMT-unmethylated (pink squares) patients before progression, and (**B**) before progression (blue circles) versus after progression (orange squares).

Next, the HUI was calculated for both meth and unmeth patients at the time of first contact, before relapse (if any), and before last contact. The median calendar period between the date of diagnosis and the first presentation at IOZK was 3.6 m (range: 2.48 m to 7.37 m) for meth patients and 3.9 m (range: 2.88 m to 11.21 m) for unmeth patients. The median HUI at first presentation was 0.906 (range: 0.257 to 1) for meth patients and 0.864 (range: 0.56 to 1) for unmeth patients. There was a correlative trend between the HUI and EQ-VAS for meth patients (Spearman r: *p* = 0.09; n = 11) and a significant correlation for unmeth patients (Spearman r: *p* < 0.0001; n = 21; one VAS data point not available). There was no correlation between HUI and KPI. The median calendar period between first contact and last measurement was 8.3 m (range: 5.9 m to 35.1 m) and 8.3 m (range: 4.6 m to 31.9 m) for meth and unmeth patients, respectively. The first median HUI values were 0.87 (range: 0.69 to 1) and 0.87 (range: 0.34 to 1), respectively, and the last HUI ranked values were 0.91 (range: 0.24 to 1) and 0.84 (range: 0.10 to 1), respectively. The median resulting calculated QALM values from the first to last appointments were 7.7 m (range: 4.3 m to 33.1 m) and 7.5 m (range: 3.1 m to 24.1 m), respectively. No statistically significant differences were observed between meth and unmeth patients. As expected, the QALM was significantly lower in a paired non-parametric Wilcoxon test compared with the calendar time (*p* < 0.0001). The overall deviation of the QALM from the calendar period was minimal for both meth and unmeth patients, indicating life lived between appointments was of good quality (Figure 9A).

Of these 33 patients, 19 showed signs of progressive disease during immunotherapy: six in the meth group and 13 in the unmeth group (Figure 9B). These patients continued IMI therapy. The median calendar period from first contact to last measurement before relapse was 6.6 m (range: 2.6 m to 22.4 m) and 5.4 m (range: 1.2 m to 14.9 m) for meth and unmeth patients, respectively, with a median observed calendar period after progressive disease of 2.4 m (range: 0.2 m to 5.5 m) and 4.0 m (range: 0.8 m to 17.0 m), respectively. The HUI values before progression were 0.90 (range: 0.78 to 1) and 0.86 (range: 0.56 to 1), respectively, compared with the median HUI values after progression of 0.90 (range: 0.24 to 1) and 0.84 (range: 0.10 to 1), respectively. The QALM values before progression amounted to a median of 5.9 m (range: 2.5 m to 20.0 m) and 3.9 m (range: 1.1 m to 13.0), respectively, and after progression, a median of 1.4 m (range: 0.2 m to 5.4 m) and 3.7 m (range: 0.7 m to 9.3 m), respectively (Figure 9B). Though a majority of patients indicated a deviation of the QALM from the calendar period, these deviations were only very minor, with a QMLL of 0.86 m overall (range: 0 to 7.8) despite relapse, indicating a remarkable stability in quality of life over the course of treatment.

## 4. Discussion

While a multiphase treatment approach is deemed a highly effective and, thus, essential part of SoC in, e.g., acute lymphoblastic leukemia treatment [46], oddly, this is lacking for more heterogeneous solid brain tumors. The rationale for separating SoC from dendritic cell (DC) immunotherapy in the IMI strategy comes from the observation that, despite the similar combination of SoC with active specific patient-derived DC therapy against GB in earlier clinical trials, only a comparatively minor improvement in OS was observed, and only in GB patients with methylated MGMT promoters [16]. The lack of effect can be attributed to the fact that DCs were administered simultaneously with TMZ [47]. In a phase IIb RCT, two patient cohorts received DC therapy either during or after their SoC regimen [48]. While terminated early and not as the primary endpoint for the comparison, patient follow-up showed a potential benefit in OS for those who received their DC vaccines after maintenance CTx [49]. DC efficacy depends on an active, strong cytotoxic T-cell population. In general, SoC RCTx affects both the tumor and the immune system [50,51], which then needs a long time to recover [52]. During the anticancer treatment phase I of IMI, SoC is combined solely with immunogenic cell death (ICD) immunotherapy. The rationale behind this is that while CTx kills tumor cells, it also alters the tumor microenvironment, weakens the immune system, and increases tumor mutational burden [50]. ICD immunotherapy is oncolytic, eliminates GCSs, and further primes the TME for other forms of immunotherapy [14,53]. The subsequent immunization phase II is planned after all chemotherapy cycles are finished. ICD immunotherapy results in a release of current tumor antigens into the bloodstream. Loading these current tumor antigens on active specific immunotherapy DCs theoretically enables better tumor immune recognition and targeting [35]. Anti-programmed cell death-ligand 1 (PD-L1, Tecentriq or atezolizumab) is given to block tumor cells from inhibiting T-cell activation and protect the DCs while triggering CD8+ T-cell [54] expansion and blocking tumor cell T-cell inactivation simultaneously [32]. In addition, anti-PD-L1 blocks TAM-induced CD8+ T-cell suppression that occurs via TRAIL [36]. The immunization phase aims to rebuild, strengthen and apply the immune system to combat the current residual tumor. This strategy should strengthen the immune system enough to overcome lingering immunosuppressive GB effects. As the tumor keeps developing over time and under treatment, a maintenance and expansion of immune protection phase III is deemed vital to provide the immune system with updated tumor recognition to match. Tumor and tumor-specific T-cell interactions increase T-cell exhaustion by upregulation of PD-1 and CTLA-4 expression on the T-cell surface [55]. Switching from atezolizumab to a combination of nivolumab or pembrolizumab with ipilimumab prevents T-cell exhaustion and reinvigorates the antitumor response [56,57]. The sensitivity of the IMI design under a global strategic treatment plan applied in a real-world setting is reflected in the significantly longer observed mPFS and mOS compared with the literature for patients with methylated and unmethylated MGMT GB.

The individualized and personalized nature of IMI resulted in overall strategic treatment plans that differed from patient to patient. Due to the treatment’s dependence on a functioning immune system, patients were only eligible to undergo IMI when they had a KPI of >60. Treatment plan differences became even more pronounced due to the highly international character of the patient population, representing 19 different countries, an indirect result of the privatized nature of the center and the treatment opportunities that can only be offered under the German “Individueller Heilversuch” [18,58]. The largest differences in IMI treatment plans were observed in ICI and repurposed drug use. Not all patients were willing or able to include ICIs in their treatment; others discontinued ICI use due to side effects. The use and choice of complementary or repurposed drugs and supplements [59,60] are primarily decided upon by the patient or local caregiver.

Remarkably, in the patient group initially reported as MGMT-promoter-methylated based on tumor tissue analysis, CCC mRNA sequencing yielded a majority of samples having elevated MGMT mRNA expression levels, while the reverse was true for the unmeth patient subgroup. This reflects the challenge of correctly determining MGMT promoter methylation status. The receiver operating characteristic analysis cut-off ranges between 9 and 21% of MGMT-methylation-positive cells [61,62], or a quantitative methylation-specific PCR (qMSP) of 0.242% [63], and both are subject to controversy. Furthermore, MGMT expression levels oscillate during the day [28] and vary throughout the tumor tissue [64]. Tumor tissue handling post-resection further affects MGMT promoter methylation status observations [65]. In addition, the high spatio-temporal heterogeneity of GB might also explain this observation, whereby the resected tissue no longer matches the tumor that has since developed [66]. These reasons, however, do not sufficiently explain why a majority, rather than an equal number, of patients displayed CCC mRNA expression levels of the opposite group. A changing pattern over time of mRNA expression in CCCs was published earlier [33]. Despite controversy and temporal inconsistency, though, MGMT methylation status remains a clear prognostic factor. Despite the observed CCC inconsistency, patients initially categorized as “MGMT-methylated” performed better in terms of mOS than MGMT-unmethylated patients. Overall, this observation underlines the importance of continued monitoring, especially for highly spatio-temporally heterogeneous tumors such as GB. It is strongly recommended not to discontinue TMZ maintenance chemotherapy based on MGMT promoter methylation status alone due to its multiple modes of action against GB cells and the TME.

As highly patient-individualized IMI was given as an adjuvant strategy embedded in SoC treatment to mostly international patients, capture of and distinction between adverse events (AEs, treatment-related or unrelated) or reactions (ARs, IMI-related) occurring between on-site treatments proved challenging. Possible ARs were discussed between physicians to establish categorization and registration. Overall, ARs were limited and reflected known side effects of treatment components. Most identified ARs indicated a lower-grade toxicity, if any occurred at all. Coincidentally, the greatest toxicity within IMI treatment was linked to ICI use, in the form of liver toxicity or diarrhea. ICI use is more commonly associated with immune-related adverse events, both in GB [17] and across different tumor types [67,68], reflecting the continued need for both continuous toxicity monitoring during treatment and the need for alternative immunotherapy development. No major toxicities were linked to IO-VAC^®^ application, reflecting the well-known safety of DC vaccination [69]. NDV administrations initially caused mild flu-like symptoms.

The date of first event (FE) was defined as the date of resection or biopsy of the primary tumor. IMI treatment started generally four months after FE, which could lead to an immortal time bias, where patients must live long enough to receive IMI. As can be seen in Figure 5 and Figure 6, patients did not relapse before the start of IMI treatment, though a few patients did relapse or died shortly after the start of treatment. As these patients did receive IMI during their diagnosed time of illness, they were included in the final analysis.

This retrospective analysis was limited by its single-center nature. However, the presented patient group was selected based on a stable, clinically oriented risk-profiling system with relevance for OS [70] and is, therefore, comparable with control groups in contemporary randomized controlled trials [10,71,72,73,74], used also as the external control arm of the DC-Vax-L study [16]. Minor differences could be noted: the median age in our patient group was slightly younger, though with a comparatively lower median KPI and a lower percentage of complete resection. Another limitation was the use of real-world data (RWD). The lack of a 1:1 control arm means RWD can, at most, reach a low level of evidence of treatment efficacy. In addition, the clinic’s private, ambulant nature and worldwide unique legal framework demand that patients have sufficient financial resources. IMI is, therefore, as of yet, only available to people of middle-to-upper wealth classes, though research indicates that it is not necessarily socioeconomic status that impacts mOS, but rather how the means available are allocated toward standard anticancer care and innovative treatment options [75,76,77,78,79,80,81,82,83]. In addition, the only requirements to initiate IMI are a KPI above 60–70 and the possibility and safety to travel for ambulatory therapy, resulting in a highly international and diverse population, with personalized IMI as the sole commonality. This resulted in a mostly Western, educated, industrialized, rich and democratic (WEIRD) population bias, as seen in psychology research; though generally, clinical studies with only localized patient groups suffer the same bias. This problem has been clearly identified in the fact that current healthcare research has been primarily conducted on a white male Western industrialized population [84,85]. Though RWD analysis cannot provide the same level of evidence as RCTs due to lack of a control population and randomization, it does provide information on the complexity of treating patients from the global populace in a real-world setting, exhibiting differences in health status, starting point, local treatment, freedom of choice and financial limitations. Patients were not excluded from treatment upon presenting with failing health or other complications, or when changing treatment strategy, which is one of the greatest assets of RWD. Any alterations resulting in major differences in national healthcare, SoC strategy, additional treatments, familial support and general lifestyle were carefully documented, but subjects remained under treatment and were included in the retrospective analysis. RWD retrospective analyses in general are expected to include patients with a lower mOS and mPFS [86] compared with clinical trial patients, as the latter represent a highly selected “treatable” minority of the actual patient population. Still, the effect of IMI is evident.

RWD analyses pave the way for general accessibility. The patients reported were treated over a ten-year period, which is unheard of when recruiting for a clinical study. In relation to immunotherapy, the uncontrolled changes in immune functioning during SoC further complicate the treatment course in spite of stratification and control at the start of an RCT [87]. Clinical trials over the last 20 years have kept coming to the same conclusion: different therapies strengthen one another and perform better as adjuvant therapies. However, due to the setup of clinical trials, testing is limited to two or three combined treatment regimens at most. More would affect the standardized protocol and cannot be combined without some personalization as to what combination, when and how much for each patient. Antitumor therapies, including immunotherapies, need to be (1) administered when the patient’s immune system reflects this to be the best time to do so; (2) administered in contemplation and rational combination with other adjuvant therapies to guide and aid overall therapy efficacy; and (3) adjusted to the individual needs of both the patient and tumor. Thus, while SoC remained unchanged throughout, scientific advances did drive IMI development, unrestricted under the German “Individueller Heilversuch”. These adaptations are what make RWD so valuable: it can be used to smooth the complicated way for individualized treatment strategies to make it to clinical trial testing while still offering opportunities for patients needing treatment today.

A major factor of therapy efficacy is its contribution to patient health-related quality of life (HRQoL). This covers anything GB- and anticancer-treatment-related that can affect patients’ physical and mental health. Despite the importance of HRQoL to patients, its monitoring is not standard practice in therapy development or patient care. If HRQoL is considered, it is often of a singular nature: a one-time assessment of patient well-being. The importance of continued monitoring of HRQoL has been well-established [88,89,90]. Repeated treating physician evaluation through the KPI most resembles longitudinal HRQoL monitoring, though being limited to patients’ visual abilities in relation to their physicians, this does not reflect HRQoL well [91,92]. To the best of our knowledge, there exists no comparative analysis to the longitudinal HRQoL study presented here. Though this means conclusions drawn must be restricted to observations on this treatment strategy, the >90% response rate among patients allows for this unprecedented demonstration of how IMI maintains HRQoL in a real-world setting. The stability of functioning and HRQoL among patients over the course of IMI treatment was remarkable. Both in the self-reported EQ-5D descriptive system as well as in self-reported EQ-VAS scores, patient experiences leveled out over the course of treatment. The observation that, of the level 5 (most severe) reports given, none were registered in the domain “Pain/Discomfort”, fits reports of GB patients under palliative care who do not report physical pain, as opposed to peripheral solid tumor patients [93]. Patients generally scored the “Usual Activity” domain well, though most level 5 answers also occurred here. This reflects a primary human tendency to continue life as much as possible in the face of adversity, only halting when there is no other choice. The remarkable stability in HUI over the course of treatment translates to a low qualitative months of life lost (QMLL) over the course of treatment, largely independent of indications of progression. Through IMI treatment, quality of life is manageable to a degree in that life gained can be actively lived.

## 5. Conclusions

We presented a retrospective analysis of 104 GB patients treated with an IMI strategy embedded in and after a SoC regimen as part of first-line treatment in a real-world setting. The ambulant strategy combined with SoC delivered in the local hospital, even in another country, is shown to be feasible without added toxicity. MGMT promoter methylation status remains a prognostic marker for OS. Despite the high level of tumor complexity, again reflected in the individualized responses to treatment between GB patients, a median OS of 24.7 m was observed under this individualized treatment strategy, a substantial improvement compared with the OS of 12 m to 15 m under SoC alone [4]. Longitudinal monitoring of selected patient health-related quality of life (HRQoL) tends towards a remarkable resilience among patients undergoing treatment. IMI can be associated with HRQoL stability despite differences in treatment strategy, tumor characteristics, and even potential relapse, though the lack of similar longitudinal analyses under different treatment strategies does not allow for conclusive comparisons. Overall, the feasibility and associated significant benefit of an IMI strategy embedded in and after SoC warrants further contemplation. Though RWD analysis does not allow for evidence generation similar to RCTs, and it remains unclear how to draw evidence out of individualized medicine, individualized medicine is felt necessarily for current patients with GB. To this end, real-world retrospective analysis could inform future research efforts on patient treatment complexity, serve to set clear testing guidelines, and bridge research and large-scale clinical adaptation.

## Figures and Tables

**Table 1:** Patient immunological status at intake, prior to start of IMI treatment *.

	Methylated			Unmethylated			Chi-Square
	L (%)	N (%)	H (%)	L (%)	N (%)	H (%)	
White blood cell count	8 (19)	31 (72)	4 (9)	5 (8)	51 (84)	45 (8)	ns
Lymphocytes	33 (77)	9 (21)	1 (2)	38 (63)	22 (37)	0 (0)	ns
Monocytes	7 (16)	32 (74)	4 (9)	6 (10)	51 (85)	3 (5)	ns
Neutrophils	5 (12)	27 (63)	11 (26)	4 (7)	46 (77)	10 (17)	ns
Eosinophils	7 (16)	32 (74)	4 (9)	7 (12)	51 (85)	2 (3)	ns
Basophils	1 (2)	39 (91)	3 (7)	0 (0)	54 (90)	6 (10)	ns
Hemoglobin	11 (26)	32 (74)	0 (0)	8 (13)	51 (84)	2 (3)	ns
Platelets	15 (35)	26 (60)	2 (5)	15 (25)	45 (74)	1 (2)	ns
T cells	36 (84)	6 (14)	1 (2)	49 (82)	11 (18)	0 (0)	ns
B cells	36 (84)	7 (16)	0 (0)	52 (87)	7 (12)	1 (2)	ns
NK cells	23 (53)	19 (44)	1 (2)	31 (52)	29 (48)	0 (0)	ns
Th2 skewing	5 (12)	25 (60)	12 (29)	3 (5)	43 (70)	15 (25)	ns
Th17 skewing	1 (3)	23 (68)	10 (29)	0 (0)	37 (66)	19 (34)	ns
Th1 skewing	7 (16)	31 (72)	5 (12)	2 (3)	50 (82)	9 (15)	ns
Th1/Th2	7 (21)	24 (71)	3 (9)	10 (18)	42 (74)	5 (9)	ns
NK cell function	26 (60)	16 (37)	1 (2)	39 (66)	20 (34)	0 (0)	ns
	**Good** **(%)**	**Moderate (%)**	**Increased (%)**	**Good (%)**	**Moderate (%)**	**Increased (%)**	
Peroxide load	11 (27)	14 (34)	16 (39)	13 (23)	15 (26)	29 (51)	ns
	**Low (%)**	**Medium (%)**	**High (%)**	**Low (%)**	**Medium (%)**	**High (%)**	
Total anti-oxidative capacity	3 (7)	6 (15)	32 (78)	1 (2)	5 (9)	51 (89)	ns
	**No CCCs (%)**	**CCC PD-L1- (%)**	**CCC PD-L1+ (%)**	**No CCCs (%)**	**CCC PD-L1- (%)**	**CCC PD-L1+ (%)**	
CCC	21 (50)	18 (43)	3 (7)	35 (58)	22 (37)	3 (5)	ns
	**No CCCs (%)**	**CCC MGMT- (%)**	**CCC MGMT+ (%)**	**No CCCs (%)**	**CCC MGMT- (%)**	**CCC MGMT+ (%)**	
CCC	21 (57)	7 (19)	9 (24)	35 (61)	13 (23)	9 (16)	

* Abbreviations: L: low, N: neutral, H: high, NK: natural killer, Th: T helper cell, CCCs: circulating cancer cells, PD-L1: programmed death-ligand 1, MGMT: O^6^-methylguanine-DNA methyltransferase, and ns: not specified. Table background colour and bold text indicate subheadings.

**Table 2:** Overview of adverse events and their severity per treatment course in the different CTCAE * domains.

CTCAE Domain	Grade < 3	Grade ≥ 3	Treatment Course
Gastrointestinal disorders	7	0	ICD + CTx (0), IO-VAC (3), ICD (4)
General disorders and administration sites conditions	9	1	ICD + CTx (3), IO-VAC (7), ICD (0)
Hepatobiliary disorders	2	1	ICD + CTx (3), IO-VAC (0), ICD (0)
Injury, poisoning and procedural complications	0	1	ICD + CTx (0), IO-VAC (1), ICD (0)
Musculoskeletal and connective tissue disorders	2	0	ICD + CTx (1), IO-VAC (0), ICD (1)
Nervous system disorders	14	5	ICD + CTx (10), IO-VAC (6), ICD (3)
Psychiatric disorders	1	0	ICD + CTx (0), IO-VAC (1), ICD (0)
Respiratory, thoracic and mediastinal disorders	0	1	ICD + CTx (0), IO-VAC (0), ICD (1)
Skin and subcutaneous tissue disorders	7	0	ICD + CTx (4), IO-VAC (1), ICD (2)
Vascular disorders	0	1	ICD + CTx (0), IO-VAC (0), ICD (1)

* Abbreviations: CTCAE: Common Terminology Criteria for Adverse Events, ICD: immunogenic cell death, and CTx: chemotherapy.

## Data Availability

The data presented in this study are available upon request from the corresponding author. The data are not publicly available due to privacy reasons.

## Abbreviations

The following abbreviations are used in this manuscript:

µg	Microgram
Acetyl CoA	Acetyl-coenzyme A
AEs	Adverse events
ARs	Adverse reactions
BBB	Blood–brain barrier
BMI	Body mass index
CBD	Cannabidoil
CCCs	Circulating cancer cells
Ccf	Circulating cell-free
CD	Cluster of differentiation
CI	Confidence interval
c-KT	C-kit proto-oncogene
CNS	Central nervous system
Cox-2	Cyclooxygenase-2
CTCAE	Common Terminology Criteria for Adverse Events
CTLA-4	Cytotoxic T-lymphocyte-associated protein 4
CTx	Chemotherapy
DC	Dendritic cell
DNA	Deoxyribonucleic acid
EDTA	Ethylenediaminetetraacetic acid
EGFR	Epidermal growth factor receptor
ERBB2	Erythroblastic oncogene B
EV	Extracellular vesicle
FE	First event
g	Gram
GB	Glioblastoma
GM-CSF	Granulocyte–macrophage colony-stimulating factor
GMP	Good manufacturing practice
GSC	Glioma stem-like cell
h	Hour
H	High
HRQoL	Health-related quality of life
HUI	Health utility index
ICD	Immunogenic cell death
ICI	Immune checkpoint inhibitor
IDH1	Isocitrate dehydrogenase 1
IFN	Interferon
IL	Interleukin
IMI	Individualized multimodal immunotherapy
Imm	Immune system
IOZK	Immune Oncology Center Cologne
kg	Kilogram
KPI	Karnofsky performance index
L	Low
LA	Last appointment
LAG-3	Lymphocyte activation gene 3
M	Molar concentration
m	Month
MDSC	Myeloid-derived suppressor cell
mEHT	Modulated electro-hyperthermia
Meth	MGMT promoter methylated
MGMT	O-6-methylguanin-DNA-methyltransferase
mL	Milliliter
MRI	Magnetic resonance imaging
mRNA	Messenger ribonucleic acid
N	Neutral
n	Number of patients; sample size
NA	Not applicable
NDV	Newcastle disease virus
NF-κB	Nuclear factor kappa-light-chain-enhancer of activated B cells
NK	Natural killer cell
ns	Not specified
OS	Overall survival
PD-1	Programmed cell death protein 1
PD-L1	Programmed cell death protein ligand 1
PFS	Progression-free survival
PH	Private hospital
PHA	Phytohemagglutinin
PMA	Phorbol 12-myristate 13-acetate
QALM	Quality-adjusted life months
QMLL	Qualitative months of life lost
R0	Complete resection
RCTx	Radiochemotherapy
ROS	Reactive oxygen species
RTx	Radiotherapy
RWD	Real-world data
S	Serum
SES	Socioeconomic status
SNH	Safety net hospital
SoC	Standard of care
STAT5	Signal transducer and activator of transcription 5
TAM	Tumor-associated macrophage
Th	T helper cells
THC	Tetrahydrocannabinol
TIM-3	T cell immunoglobulin and mucin domain-containing protein 3
TL	Tumor lysate
TME	Tumor microenvironment
TMZ	Temozolomide
TOS/TOC	Total oxidant status/total oxidant capacity
TRAIL	Tumor necrosis factor-related apoptosis-inducing ligand
TTFs	Tumor-treating fields
U	Unit
Unmeth	MGMT promoter unmethylated
VAF	Variant allele frequency
VAS	Visual analog scale
wt	Wild type
WT1	Wilms tumor 1 gene
y	Year

## References

[1] Johnson D.R., O’Neill B.P. (2012). Glioblastoma Survival in the United States before and during the Temozolomide Era. J. Neurooncol..

[2] Antonopoulos M., Gool S.W.V., Dionysiou D., Graf N., Stamatakos G. (2019). Immune Phenotype Correlates with Survival in Patients With GBM Treated With Standard Temozolomide-Based Therapy and Immunotherapy. Anticancer Res..

[3] Weller M., Le Rhun E., Preusser M., Tonn J.-C., Roth P. (2019). How We Treat Glioblastoma. ESMO Open.

[4] Weller M., Cloughesy T., Perry J.R., Wick W. (2013). Standards of Care for Treatment of Recurrent Glioblastoma—Are We There Yet?. Neuro-Oncology.

[5] Ostrom Q.T., Gittleman H., Farah P., Ondracek A., Chen Y., Wolinsky Y., Stroup N.E., Kruchko C., Barnholtz-Sloan J.S. (2013). CBTRUS Statistical Report: Primary Brain and Central Nervous System Tumors Diagnosed in the United States in 2006–2010. Neuro-Oncology.

[6] Ostrom Q.T., Gittleman H., Liao P., Vecchione-Koval T., Wolinsky Y., Kruchko C., Barnholtz-Sloan J.S. (2017). CBTRUS Statistical Report: Primary Brain and Other Central Nervous System Tumors Diagnosed in the United States in 2010–2014. Neuro-Oncology.

[7] Ostrom Q.T., Cioffi G., Waite K., Kruchko C., Barnholtz-Sloan J.S. (2021). CBTRUS Statistical Report: Primary Brain and Other Central Nervous System Tumors Diagnosed in the United States in 2014–2018. Neuro-Oncology.

[8] Price M., Ballard C.A.P., Benedetti J.R., Kruchko C., Barnholtz-Sloan J.S., Ostrom Q.T. (2025). CBTRUS Statistical Report: Primary Brain and Other Central Nervous System Tumors Diagnosed in the United States in 2018–2022. Neuro-Oncology.

[9] Franceschi E., Ermani M., Bartolini S., Bartolotti M., Poggi R., Tallini G., Marucci G., Fioravanti A., Tosoni A., Agati R. (2015). Post Progression Survival in Glioblastoma: Where Are We?. J. Neurooncol..

[10] Stupp R., Taillibert S., Kanner A., Read W., Steinberg D.M., Lhermitte B., Toms S., Idbaih A., Ahluwalia M.S., Fink K. (2017). Effect of Tumor-Treating Fields Plus Maintenance Temozolomide vs Maintenance Temozolomide Alone on Survival in Patients With Glioblastoma: A Randomized Clinical Trial. JAMA.

[11] Riegel D.C., Bureau B.L., Conlon P., Chavez G., Connelly J.M. (2025). Long-Term Survival, Patterns of Progression, and Patterns of Use for Patients with Newly Diagnosed Glioblastoma Treated with or without Tumor Treating Fields (TTFields) in a Real-World Setting. J. Neurooncol..

[12] Szasz A.M., Alvarez E.E.A., Fiorentini G., Herold M., Herold Z., Sarti D., Dank M. (2023). Meta-Analysis of Modulated Electro-Hyperthermia and Tumor Treating Fields in the Treatment of Glioblastomas. Cancers.

[13] Giovannoni F., Strathdee C.A., Faust Akl C., Andersen B.M., Li Z., Lee H.-G., Torti M.F., Rone J.M., Duart-Abadia P., Molgora M. (2025). Retargeted Oncolytic Viruses Engineered to Remodel the Tumor Microenvironment for Glioblastoma Immunotherapy. Nat. Cancer.

[14] Schirrmacher V., van Gool S., Stuecker W. (2022). Counteracting Immunosuppression in the Tumor Microenvironment by Oncolytic Newcastle Disease Virus and Cellular Immunotherapy. Int. J. Mol. Sci..

[15] Bota D.A., Piccioni D.E., Duma C.M., Kesari S., Carrillo J.A., LaRocca R.V., Aiken R.D., Taylor T.H., Abedi M., Robles R.M. (2025). Phase 2 Trial of Personal Dendritic Cell Vaccines in Newly Diagnosed Glioblastoma: 3-Year Follow-up and Correlations with Survival. Hum. Vaccines Immunother..

[16] Liau L.M., Ashkan K., Brem S., Campian J.L., Trusheim J.E., Iwamoto F.M., Tran D.D., Ansstas G., Cobbs C.S., Heth J.A. (2023). Association of Autologous Tumor Lysate-Loaded Dendritic Cell Vaccination With Extension of Survival Among Patients With Newly Diagnosed and Recurrent Glioblastoma: A Phase 3 Prospective Externally Controlled Cohort Trial. JAMA Oncol..

[17] Gómez E.G., Morales M.A.M., San-Juan D., Romero-Valencia J., Mendez D.L.R., Hernandez D.O.L., Pizarro M.M., Narvaez E.R.B., Pelayo-Salazar M.E., Jimenez S.M. (2026). Efficacy and Safety of Immune Checkpoint Inhibitors and mTOR Inhibitors as Targeted Therapy for Glioblastoma: A Systematic Review and Meta-Analysis of Randomized Clinical Trials. Neurosurg. Rev..

[18] Van Gool S.W., Van de Vliet P., Kampers L.F.C., Kosmal J., Sprenger T., Reich E., Schirrmacher V., Stuecker W. (2023). Methods behind Oncolytic Virus-Based DC Vaccines in Cancer: Toward a Multiphase Combined Treatment Strategy for Glioblastoma (GBM) Patients. Methods in Cell Biology.

[19] Kampers L.F.C., Metselaar D.S., Vinci M., Scirocchi F., Veldhuijzen van Zanten S., Eyrich M., Biassoni V., Hulleman E., Karremann M., Stücker W. (2025). The Complexity of Malignant Glioma Treatment. Cancers.

[20] Saxena K., Jolly M.K. (2019). Acute vs. Chronic vs. Cyclic Hypoxia: Their Differential Dynamics, Molecular Mechanisms, and Effects on Tumor Progression. Biomolecules.

[21] Zhou W., Wahl D.R. (2019). Metabolic Abnormalities in Glioblastoma and Metabolic Strategies to Overcome Treatment Resistance. Cancers.

[22] Zhang X., Zhao L., Zhang H., Zhang Y., Ju H., Wang X., Ren H., Zhu X., Dong Y. (2022). The Immunosuppressive Microenvironment and Immunotherapy in Human Glioblastoma. Front. Immunol..

[23] Lerouge L., Ruch A., Pierson J., Thomas N., Barberi-Heyob M. (2024). Non-Targeted Effects of Radiation Therapy for Glioblastoma. Heliyon.

[24] Li H., Du Z., Lu H., Xi J., Hu H. (2025). A Novel Mucosa-Associated Lymphoid Tissue Translocation Gene 1-Related Immune Prognostic Signature and Targeted Drug Screening for Glioblastoma. Brain Circ..

[25] Venkataramani V., Tanev D.I., Strahle C., Studier-Fischer A., Fankhauser L., Kessler T., Körber C., Kardorff M., Ratliff M., Xie R. (2019). Glutamatergic Synaptic Input to Glioma Cells Drives Brain Tumour Progression. Nature.

[26] Amiama-Roig A., Verdugo-Sivianes E.M., Carnero A., Blanco J.-R. (2022). Chronotherapy: Circadian Rhythms and Their Influence in Cancer Therapy. Cancers.

[27] Quist M., van Os M., van Laake L.W., Bovenschen N., Crnko S. (2024). Integration of Circadian Rhythms and Immunotherapy for Enhanced Precision in Brain Cancer Treatment. eBioMedicine.

[28] Gonzalez-Aponte M.F., Huang Y., Leidig W.A., Simon T., Butt O.H., Ruben M.D., Kim A.H., Rubin J.B., Herzog E.D., Walch O.J. (2025). Circadian Variation in MGMT Promoter Methylation and Expression Predicts Sensitivity to Temozolomide in Glioblastoma. J. Neurooncol..

[29] Ganesh N., Saleem A., Babu R., Kochuthakidiyel Suresh P. (2025). Gut Brain Axis and Gut Microbiome in Glioblastoma. Associations, Treatment and Outcomes. Med. Microecol..

[30] Chen R., Su J., Dong X., Tang X., Yang Y., Li Q. (2026). Entinostat and Tucidinostat Potentiate Temozolomide Response in Glioblastoma Models. Pharmazie.

[31] Besson J.-M., Chavanieu A., Nadaradjane A., Cartron P.-F., Lopez M. (2026). From AlphaFold to Site-Selective Inhibition of DNA Methylation in Glioblastoma Multiforme. RSC Med. Chem..

[32] Peng Q., Qiu X., Zhang Z., Zhang S., Zhang Y., Liang Y., Guo J., Peng H., Chen M., Fu Y.-X. (2020). PD-L1 on Dendritic Cells Attenuates T Cell Activation and Regulates Response to Immune Checkpoint Blockade. Nat. Commun..

[33] Van Gool S.W., Makalowski J., Van de Vliet P., Van Gool S., Sprenger T., Schirrmacher V., Stuecker W. (2023). Individualized Multimodal Immunotherapy for Adults with IDH1 Wild-Type GBM: A Single Institute Experience. Cancers.

[34] Louis D.N., Perry A., Wesseling P., Brat D.J., Cree I.A., Figarella-Branger D., Hawkins C., Ng H.K., Pfister S.M., Reifenberger G. (2021). The 2021 WHO Classification of Tumors of the Central Nervous System: A Summary. Neuro-Oncology.

[35] Garg A.D., Vandenberk L., Koks C., Verschuere T., Boon L., Van Gool S.W., Agostinis P. (2016). Dendritic Cell Vaccines Based on Immunogenic Cell Death Elicit Danger Signals and T Cell–Driven Rejection of High-Grade Glioma. Sci. Transl. Med..

[36] Sprooten J., Vanmeerbeek I., Datsi A., Govaerts J., Naulaerts S., Laureano R.S., Borràs D.M., Calvet A., Malviya V., Kuballa M. (2024). Lymph Node and Tumor-Associated PD-L1^+^ Macrophages Antagonize Dendritic Cell Vaccines by Suppressing CD8^+^ T Cells. Cell Rep. Med..

[37] Kleef R., Nagy R., Baierl A., Bacher V., Bojar H., McKee D.L., Moss R., Thoennissen N.H., Szász M., Bakacs T. (2021). Low-Dose Ipilimumab plus Nivolumab Combined with IL-2 and Hyperthermia in Cancer Patients with Advanced Disease: Exploratory Findings of a Case Series of 131 Stage IV Cancers—A Retrospective Study of a Single Institution. Cancer Immunol. Immunother..

[38] Paul S., Sa G. (2021). Curcumin as an Adjuvant to Cancer Immunotherapy. Front. Oncol..

[39] Gersey Z.C., Rodriguez G.A., Barbarite E., Sanchez A., Walters W.M., Ohaeto K.C., Komotar R.J., Graham R.M. (2017). Curcumin Decreases Malignant Characteristics of Glioblastoma Stem Cells via Induction of Reactive Oxygen Species. BMC Cancer.

[40] Karnofsky D.A., Burchenal J.H., MacLeod C.M. (1949). Evaluation of Chemotherapeutic Agents.

[41] Euroqol *EQ-5D-5L*; EuroQol. https://euroqol.org/information-and-support/euroqol-instruments/eq-5d-5l/.

[42] Bitar M., Boldt A., Freitag M.-T., Gruhn B., Köhl U., Sack U. (2019). Evaluating STAT5 Phosphorylation as a Mean to Assess T Cell Proliferation. Front. Immunol..

[43] Kandarian F., Sunga G.M., Arango-Saenz D., Rossetti M. (2017). A Flow Cytometry-Based Cytotoxicity Assay for the Assessment of Human NK Cell Activity. JoVE (J. Vis. Exp.).

[44] Hallermayr A., Keßler T., Fujera M., Liesfeld B., Bernstein S., von Ameln S., Schanze D., Steinke-Lange V., Pickl J.M.A., Neuhann T.M. (2023). Impact of cfDNA Reference Materials on Clinical Performance of Liquid Biopsy NGS Assays. Cancers.

[45] US Department of Health and Human Services (2017). Common Terminology Criteria for Adverse Events (CTCAE) v5.0. https://dctd.cancer.gov/research/ctep-trials/for-sites/adverse-events/ctcae-v5-5x7.pdf.

[46] Würthwein G., Siebel C., Lanvers-Kaminsky C., Smisek P., Nath C.E., Matteo C., Rizzari C., Schrappe M., Boos J., The AIEOP-BFM ALL 2009 Asparaginase Working Party (2025). PEGylated Asparaginase in Children with Acute Lymphoblastic Leukemia Treated within the AIEOP-BFM ALL 2009 Trial: Population Pharmacokinetics and Drug Exposure. Eur. J. Drug Metab. Pharmacokinet..

[47] Van Gool S.W., Makalowski J., Kampers L.F.C., Van de Vliet P., Sprenger T., Schirrmacher V., Stücker W. (2023). Dendritic Cell Vaccination for Glioblastoma Multiforme Patients: Has a New Milestone Been Reached?. Transl. Cancer Res..

[48] Gool V., Willy S. (2015). Brain Tumor Immunotherapy: What Have We Learned so Far?. Front. Oncol..

[49] Dejaegher J., Solie L., Hunin Z., Sciot R., Capper D., Siewert C., Van Cauter S., Wilms G., van Loon J., Ectors N. (2021). DNA Methylation Based Glioblastoma Subclassification Is Related to Tumoral T-Cell Infiltration and Patient Survival. Neuro-Oncology.

[50] Sharma A., Jasrotia S., Kumar A. (2024). Effects of Chemotherapy on the Immune System: Implications for Cancer Treatment and Patient Outcomes. Naunyn. Schmiedebergs Arch. Pharmacol..

[51] Dutoit V., Philippin G., Widmer V., Marinari E., Vuilleumier A., Migliorini D., Schaller K., Dietrich P.-Y. (2020). Impact of Radiochemotherapy on Immune Cell Subtypes in High-Grade Glioma Patients. Front. Oncol..

[52] Kang D.-H., Weaver M.T., Park N.-J., Smith B., McArdle T., Carpenter J. (2009). Significant Impairment in Immune Recovery After Cancer Treatment. Nurs. Res..

[53] Fucikova J., Kepp O., Kasikova L., Petroni G., Yamazaki T., Liu P., Zhao L., Spisek R., Kroemer G., Galluzzi L. (2020). Detection of Immunogenic Cell Death and Its Relevance for Cancer Therapy. Cell Death Dis..

[54] Franken A., Bila M., Mechels A., Kint S., Dessel J.V., Pomella V., Vanuytven S., Philips G., Bricard O., Xiong J. (2024). CD4^+^ T Cell Activation Distinguishes Response to Anti-PD-L1+anti-CTLA4 Therapy from Anti-PD-L1 Monotherapy. Immunity.

[55] Woroniecka K., Chongsathidkiet P., Rhodin K., Kemeny H., Dechant C., Farber S.H., Elsamadicy A.A., Cui X., Koyama S., Jackson C. (2018). T-Cell Exhaustion Signatures Vary with Tumor Type and Are Severe in Glioblastoma. Clin. Cancer Res..

[56] Wang K., Coutifaris P., Brocks D., Wang G., Azar T., Solis S., Nandi A., Anderson S., Han N., Manne S. (2024). Combination Anti-PD-1 and Anti-CTLA-4 Therapy Generates Waves of Clonal Responses That Include Progenitor-Exhausted CD8^+^ T Cells. Cancer Cell.

[57] Rotte A. (2019). Combination of CTLA-4 and PD-1 Blockers for Treatment of Cancer. J. Exp. Clin. Cancer Res..

[58] Huber F. (2015). Individueller Heilversuch Und Klinisches Experiment. Ph.D. Thesis.

[59] Le Rhun E., Devos P., Bourg V., Darlix A., Lorgis V., Ahle G., Boone M., Taillandier L., Curtit E., Gras L. (2019). Complementary and Alternative Medicine Use in Glioma Patients in France. J. Neurooncol..

[60] Eisele G., Roelcke U., Conen K., Huber F., Weiss T., Hofer S., Heese O., Westphal M., Hertler C., Roth P. (2019). Complementary and Alternative Medicine Use by Glioma Patients in Switzerland. Neuro-Oncol. Pract..

[61] Nguyen N., Redfield J., Ballo M., Michael M., Sorenson J., Dibaba D., Wan J., Ramos G.D., Pandey M. (2021). Identifying the Optimal Cutoff Point for MGMT Promoter Methylation Status in Glioblastoma. CNS Oncol..

[62] Brigliadori G., Foca F., Dall’Agata M., Rengucci C., Melegari E., Cerasoli S., Amadori D., Calistri D., Faedi M. (2016). Defining the Cutoff Value of MGMT Gene Promoter Methylation and Its Predictive Capacity in Glioblastoma. J. Neurooncol..

[63] Huseyinoglu Z., Uysal E., Inan M.A., Demirci T., Gokay G., Aras F.K., Erdamar S., Ozduman K., Pamir M.N., Danyeli A.E. (2025). Optimal qMSP Cutoff Value for MGMT Promoter Methylation in Glioblastoma and Its Validation for Clinical Significance. BMC Cancer.

[64] Della Puppa A., Persano L., Masi G., Rampazzo E., Sinigaglia A., Pistollato F., Denaro L., Barzon L., Palù G., Basso G. (2012). MGMT Expression and Promoter Methylation Status May Depend on the Site of Surgical Sample Collection within Glioblastoma: A Possible Pitfall in Stratification of Patients?. J. Neurooncol..

[65] Hamilton M.G., Roldán G., Magliocco A., McIntyre J.B., Parney I., Easaw J.C. (2011). Determination of the Methylation Status of MGMT in Different Regions within Glioblastoma Multiforme. J. Neurooncol..

[66] Klughammer J., Kiesel B., Roetzer T., Fortelny N., Nemc A., Nenning K.-H., Furtner J., Sheffield N.C., Datlinger P., Peter N. (2018). The DNA Methylation Landscape of Glioblastoma Disease Progression Shows Extensive Heterogeneity in Time and Space. Nat. Med..

[67] Wang B., Hao X., Yan J., Li X., Zhao M., Han T. (2024). A Bibliometric Analysis of Immune-Related Adverse Events in Cancer Patients and a Meta-Analysis of Immune-Related Adverse Events in Patients with Hepatocellular Carcinoma. J. Transl. Intern. Med..

[68] Herrmann J., Barac A., Carver J., Cheng R.K., Daniele A., Dent S., Deych E., Lee D.H., Lenihan D., Leong D.P. (2026). Immune Checkpoint Inhibitor–Associated Cardiovascular Toxic Effects: International Cardio-Oncology Society Position Statement. JAMA Oncol..

[69] Lv L., Huang J., Xi H., Zhou X. (2020). Efficacy and Safety of Dendritic Cell Vaccines for Patients with Glioblastoma: A Meta-Analysis of Randomized Controlled Trials. Int. Immunopharmacol..

[70] Stupp R., Hegi M.E., Mason W.P., van den Bent M.J., Taphoorn M.J., Janzer R.C., Ludwin S.K., Allgeier A., Fisher B., Belanger K. (2009). Effects of Radiotherapy with Concomitant and Adjuvant Temozolomide versus Radiotherapy Alone on Survival in Glioblastoma in a Randomised Phase III Study: 5-Year Analysis of the EORTC-NCIC Trial. Lancet Oncol..

[71] Weller M., Butowski N., Tran D.D., Recht L.D., Lim M., Hirte H., Ashby L., Mechtler L., Goldlust S.A., Iwamoto F. (2017). Rindopepimut with Temozolomide for Patients with Newly Diagnosed, EGFRvIII-Expressing Glioblastoma (ACT IV): A Randomised, Double-Blind, International Phase 3 Trial. Lancet Oncol..

[72] Gilbert M.R., Dignam J.J., Armstrong T.S., Wefel J.S., Blumenthal D.T., Vogelbaum M.A., Colman H., Chakravarti A., Pugh S., Won M. (2014). A Randomized Trial of Bevacizumab for Newly Diagnosed Glioblastoma. N. Engl. J. Med..

[73] Gilbert M.R., Wang M., Aldape K.D., Stupp R., Hegi M.E., Jaeckle K.A., Armstrong T.S., Wefel J.S., Won M., Blumenthal D.T. (2013). Dose-Dense Temozolomide for Newly Diagnosed Glioblastoma: A Randomized Phase III Clinical Trial. J. Clin. Oncol..

[74] Wen P.Y., Reardon D.A., Armstrong T.S., Phuphanich S., Aiken R.D., Landolfi J.C., Curry W.T., Zhu J.-J., Glantz M., Peereboom D.M. (2019). A Randomized Double-Blind Placebo-Controlled Phase II Trial of Dendritic Cell Vaccine ICT-107 in Newly Diagnosed Patients with Glioblastoma. Clin. Cancer Res..

[75] Stubbs N.M., Garner J.A., Akinwunmi-Williams T., Jani J., Ngo S., Ma T., Robinson E., Jackson T., Olson J., Huntoon K. (2025). Financial Toxicity in High-Grade Glioma Resection: A Retrospective Analysis. World Neurosurg..

[76] Bower A., Hsu F.-C., Weaver K.E., Yelton C., Merrill R., Wicks R., Soike M., Hutchinson A., McTyre E., Laxton A. (2020). Community Economic Factors Influence Outcomes for Patients with Primary Malignant Glioma. Neuro-Oncol. Pract..

[77] Sun Y., Estevez-Ordonez D., Atchley T.J., Nabors B., Markert J.M. (2025). The Association of Neighborhood-Level Deprivation with Glioblastoma Outcomes: A Single Center Cohort Study. J. Neurooncol..

[78] Wijaya J.H., Hulou S., Lucke-Wold B., Gillam V W.B., Lamprecht C.B., Ruchika F., Schneider B., Foster D., Abolfazli P., Quintero-Consuegra M.D. (2025). Socioeconomic Status as a Determinant of Survival in Glioblastoma: A Systematic Review and Meta-Analysis. Neurosurg. Rev..

[79] Serchen J., Johnson D., Cline K., Hilden D., Algase L.F., Silberger J.R., Watkins C., Health and Public Policy Committee and the Medical Practice and Quality Committee of the American College of Physicians (2025). Improving Health and Health Care in Rural Communities: A Position Paper from the American College of Physicians. Ann. Intern. Med..

[80] Sousa M.D., Whitley L.A. (2026). The United States Health Care Crisis, Private Health Savings Accounts, and Bankruptcy.

[81] Kasl R.A., Brinson P.R., Chambless L.B. (2016). Socioeconomic Status Does Not Affect Prognosis in Patients with Glioblastoma Multiforme. Surg. Neurol. Int..

[82] Demetz M., Krigers A., Klingenschmid J., Thomé C., Freyschlag C.F., Kerschbaumer J. (2025). Socioeconomic Influences on Survival Outcome in Idh-Wildtype Glioma Patients: Examining the Role of Age, Education, and Lifestyle Factors. Acta Neurochir..

[83] Wang T., Pham A., Yoo S., Attenello F.J., Jennelle R., Wagle N., Chang E.L., Zada G. (2020). Identifying Disparities in Care in Treating Glioblastoma: A Retrospective Cohort Study of Patients Treated at a Safety-Net Versus Private Hospital Setting. World Neurosurg..

[84] Lilley C.M., Arnason T., Feffer M., Moore M., Bernstein S.A., Mirza K.M. (2026). Testing Bias: The Hidden Influence of Race, Gender, and Identity in Medical Assessment. Acad. Pathol..

[85] Debay V., Moghrabi M., Barriga S., Goldfarb M. (2026). Characteristics of Family Participants in Randomized ICU Clinical Trials: A Systematic Review. Intensive Crit. Care Nurs..

[86] Skaga E., Skretteberg M.A., Johannesen T.B., Brandal P., Vik-Mo E.O., Helseth E., Langmoen I.A. (2021). Real-World Validity of Randomized Controlled Phase III Trials in Newly Diagnosed Glioblastoma: To Whom Do the Results of the Trials Apply?. Neuro-Oncol. Adv..

[87] Van Gool S.W., Makalowski J., Fiore S., Sprenger T., Prix L., Schirrmacher V., Stuecker W. (2021). Randomized Controlled Immunotherapy Clinical Trials for GBM Challenged. Cancers.

[88] Buiting H.M., Olthuis G. (2020). Importance of Quality-of-Life Measurement Throughout the Disease Course. JAMA Netw. Open.

[89] Coomans M.B., Dirven L., Aaronson N., Baumert B.G., van den Bent M., Bottomley A., Brandes A.A., Chinot O., Coens C., Gorlia T. (2022). Factors Associated with Health-Related Quality of Life (HRQoL) Deterioration in Glioma Patients during the Progression-Free Survival Period. Neuro-Oncology.

[90] Haslam A., Herrera-Perez D., Gill J., Prasad V. (2020). Patient Experience Captured by Quality-of-Life Measurement in Oncology Clinical Trials. JAMA Netw. Open.

[91] Mackworth N., Fobair P., Prados M.D. (1992). Quality of Life Self-Reports from 200 Brain Tumor Patients: Comparisons with Karnofsky Performance Scores. J. Neurooncol..

[92] Stedman M.R., Watford D.J., Chertow G.M., Tan J.C. (2021). Karnofsky Performance Score—Failure to Thrive as a Frailty Proxy?. Transplant. Direct.

[93] Giammalva G.R., Iacopino D.G., Azzarello G., Gaggiotti C., Graziano F., Gulì C., Pino M.A., Maugeri R. (2018). End-of-Life Care in High-Grade Glioma Patients. The Palliative and Supportive Perspective. Brain Sci..

